# Less is more: natural variation disrupting a miR172 gene at the *di* locus underlies the recessive double-flower trait in peach (*P. persica* L. Batsch)

**DOI:** 10.1186/s12870-022-03691-w

**Published:** 2022-07-04

**Authors:** Marco Cirilli, Laura Rossini, Remo Chiozzotto, Irina Baccichet, Francesco Elia Florio, Angelo Mazzaglia, Silvia Turco, Daniele Bassi, Stefano Gattolin

**Affiliations:** 1 Department of Agricultural and Environmental Sciences (DISAA), University of Milan, 20133 Milan, Italy; 2 DAFNE Department - University of Tuscia, 01100 Viterbo, Italy; 3 CNR - National Research Council of Italy, Institute of Agricultural Biology and Biotechnology (IBBA), 20133 Milan, Italy

**Keywords:** *Prunus persica* L. Batsch, Ornamental traits, Petal number, miRNA, miR172

## Abstract

**Background:**

With the domestication of ornamental plants, artificial selective pressure favored the propagation of mutations affecting flower shape, and double-flower varieties are now readily available for many species. In peach two distinct loci control the double-flower phenotype: the dominant *Di2* locus, regulated by the deletion of the binding site for miR172 in the euAP2 *PETALOSA* gene *Prupe.6G242400*, and the recessive *di* locus, of which the underlying factor is still unknown.

**Results:**

Based on its genomic location a candidate gene approach was used to identify genetic variants in a diverse panel of ornamental peach accessions and uncovered three independent mutations in *Prupe.2G237700*, the gene encoding the transcript for microRNA *miR172d*: a ~5.0 Kb LTR transposable element and a ~1.2 Kb insertion both positioned upstream of the sequence encoding the *pre-miR172d* within the transcribed region of *Prupe.2G237700*, and a ~9.5 Kb deletion encompassing the whole gene sequence. qRT-PCR analysis confirmed that expression of *pre-miR172d* was abolished in *di/di* genotypes homozygous for the three variants.

**Conclusions:**

Collectively, *PETALOSA* and the mutations in micro-RNA *miR172d* identified in this work provide a comprehensive collection of the genetic determinants at the base of the double-flower trait in the peach germplasms.

**Supplementary Information:**

The online version contains supplementary material available at 10.1186/s12870-022-03691-w.

## Background

Peach (*Prunus persica* L.) is a deciduous tree species belonging to the Rosaceae family widely grown in temperate regions of both the Northern and Southern hemispheres. It originates from China, where the domestication process could have started as early as 7,500 years ago [1]. Between 3000 and 2000 years ago the cultivation of this crop spread through the Asian continent, to the Middle East and Europe, and was more recently brought to the Americas by Spanish and Portuguese explorers [2]. Since a long time, peach is appreciated as an arboreal ornamental plant for the flamboyance of its springtime blooms, particularly sought-after in Eastern countries such as Japan and China, where it is a symbol of luck and happiness. Ornamental peaches are characterized by a huge variability for traits related to tree growth habit, flower and leaf colors, flower shape and type. Growth habits vary from upright to dwarf, weeping, and fastigiate [3]. Flower color is usually pink but in different cultivars it ranges from white to magenta or red, occasionally displaying bicolor or tricolor stripes in ‘variegated’ varieties. While the cultivated peach typically bears flowers with five sepals, five petals, many stamens and one pistil, ‘Flore pleno’ or ‘Double flower’ varieties are instead characterized by extra flower organs, particularly petals (from 10 – 25 in ‘semi-double’ to more than 30 – 40 in double cultivars) [4]. The degree of doubleness and differences in petal shapes concurrently result in different flower types, recognized as ‘Mei-flower’ (rounded), ‘Rose’ (cup-shaped), ‘Peony’ (petaloid stamens) and ‘Chrysanthemum’ (non-showy narrow petals) types [3]. Like for other core eudicots, the flower meristem gives rise to four concentric whorls that differentiate, from the outside to the inside, into sepals and petals, the two whorls that define the outer perianth, and into stamens and a pistil, the reproductive organs. To explain the floral patterning, the so-called ABCDE model was devised using knowledge built from homoeotic mutants in model species [5–8] and has helped research in the topic for three decades now. The model divides different transcription factors into classes (A, B, C, D, E) based on their function, and the combination of different classes defines the ontogeny of each floral whorl. D-function genes are responsible for ovule identity [9], and the E-class genes act throughout the floral meristem, while A-, B- and C-class genes exert their action in adjacent whorls. A- and E- gene activity determines the formation of sepals in the first whorl, while the overlapping of their action with that of B-class genes leads to the formation of petals in whorl two. In whorl three the presence of B-, C- and E- functions is responsible of the differentiation of stamens, while the exclusive presence of C- and E-gene activity allows for carpel differentiation in the central whorl. In addition to their organ specification activity, the A-class and C-class genes were also found to mutually repress each other and play a cadastral role in maintaining each other’s activity in the whorl they contribute to shape. Nearly all these floral homoeotic genes encode MADS-domain transcription factors, with the C-function represented by *AGAMOUS* (*AG*) in *Arabidopsis* thaliana [5]. The A-class genes are instead members of the euAP2 family, transcription factors characterized by two AP2 DNA-binding and EAR-repressor domains, and containing a binding site for miR172 [10–12]. In Arabidopsis the promoter of the euAP2 gene APETALA2 (AP2) gene is active in all whorls [13], but accumulation of the resulting protein in whorl three and four is modulated by the complementary expression of miR172. Spatial and temporal patterns of expression appear to be crucial in fine-tuning organ identity, also beyond the ABCDE model: for example a partial overlap of AP2 and miR172 at whorl three during early flower development, causing a transient co-existence of AP2 and AG activities, was proposed to be crucial for stamen development [14]. Alteration at the miR172-binding site of AP2 genes resulted in phenotypes like over-proliferation of third-whorl organs, conversion of stamens to petals and even loss of floral determinacy, depending on the promoter used [13–15].

The first study about peach flower doubleness (hereafter double-flower trait, DF) traces back to Lammerts (1945) in the early 40s and was based on analysis of some F_2_ progenies from the DF cultivars ‘Early Double Pink’ and ‘Early Double Red’, bearing 12-15 and 18-24 supernumerary petals, respectively. Based on the segregation pattern, petal number was tentatively explained by the presence of three genetic factors: *d1*, completely recessive, *dm1* and *dm2* incompletely recessive. Trees homozygous for *d1d1* have only 1 to 5 extra petals, *d1d1 dm1dm1* 10 to 16 extra-petals (as in 'Early Double Pink') while *d1d1 dm1dm1 dm2dm2* 15 to 24 extra-petals (as in 'Early Double Red' and 'Peppermint Stick'). The presence of a recessive inherited DF trait has been later confirmed in other materials, such as progenies issued from 'Pillar' accession (also known as NJ Pillar) and ‘Helen Borchers' [16]. The *di* locus was strictly associated to the fastigiated habitus (i.e. columnar growth habit) controlled by the *Br*/*br* locus in a genetic map built from an F2 ‘NC174RL’ x ‘Pillar’ progeny [17, 18] and was later assigned to linkage group 2 [19]. More recently, genome-wide association studies detected signal peaks on chromosome 2 at around 21,5 and 24,7 Mb (based on the peach V2.0 reference genome) [20, 21], not far from the candidate gene for *Br* habitus (i.e. TILLER ANGLE CONTROL 1, *Prupe.2G194000.1*), found at position 23.2 Mb [22]. Nevertheless, the genetic determinant of the *di* locus has not yet been identified. Another locus conferring DF trait harbors a single dominant gene (Di2/di2, double flower/single flower), first described by Beckman *et al.* [23] and also further confirmed in genome-wide association studies [20, 21]. The *Di2* locus has been fine-mapped on the distal end of chromosome 6 in two F2 progenies from ‘NJ Weeping’ (an accession derived from ‘Red Weeping’, or probably the same genotype) x ‘Bounty’ and ‘Weeping Flower Peach’ x ‘Pamirskij 5’ crosses, where a strong candidate causal mutation was proposed in the A-function TOE-type gene *Prupe.6G242400* [24], encoding a member of the euAP2 family of transcription factors. The mutation consisted in the deletion of the three-prime end of coding sequence, predicted to result in a shorter transcript deprived of the miR172 recognition sequence. The putative transcription factor resulting from the mutated allele was proposed to retain all the known key domains required for its function, but escape the spatial/temporal repression normally granted by miR172. The phenotype was consistent with an increase of meristem size and increased number of floral organs, possibly as consequence of an antagonizing role of this euAP2 on AG-like factors activity in the center of the meristem and impairment of their negative regulation on WUSCHEL [13, 25, 26]. Similar mutations in genes belonging to a family of orthologues to *Prupe.6G242400*, collectively identified as *PETALOSA* (*PET*), were found to consistently lead to dominant double-flower phenotypes in a variety of eudicots (rose species, carnation and petunia) [27]. Furthermore, CrispR-CAS9 induced mutations directed to the miR172 recognition sequence of a *PET* gene where also shown to lead to double flowers in tobacco [27].

Building on the knowledge of the genomic location of locus *di*, the molecular determinants of the recessive inherited double-flower trait were investigated through a candidate gene approach in a panel of ornamental peach accessions from different worldwide collections. Three distinct mutations at the microRNA *miR172d*-encoding transcript *Prupe.2G237700* (named *di*^*1*^, *di*^*2*^ and *di*^*Δ*^) resulting in *pre-miR172d* miss-expression were identified as likely causes of the double-flower phenotype. Sequencing-based genotyping of ornamental accessions in available germplasm collections from Eastern countries revealed the presence of *di*^*1*^ and *di*^*2*^ allelic variants and the absence of *di*^*Δ*^, probably originated as an independent mutation after the peach cultivation spread in Europe.

## Results

### The *di* locus maps with a gene transcribing a miR172 precursor sequence

**Fig. 1:**
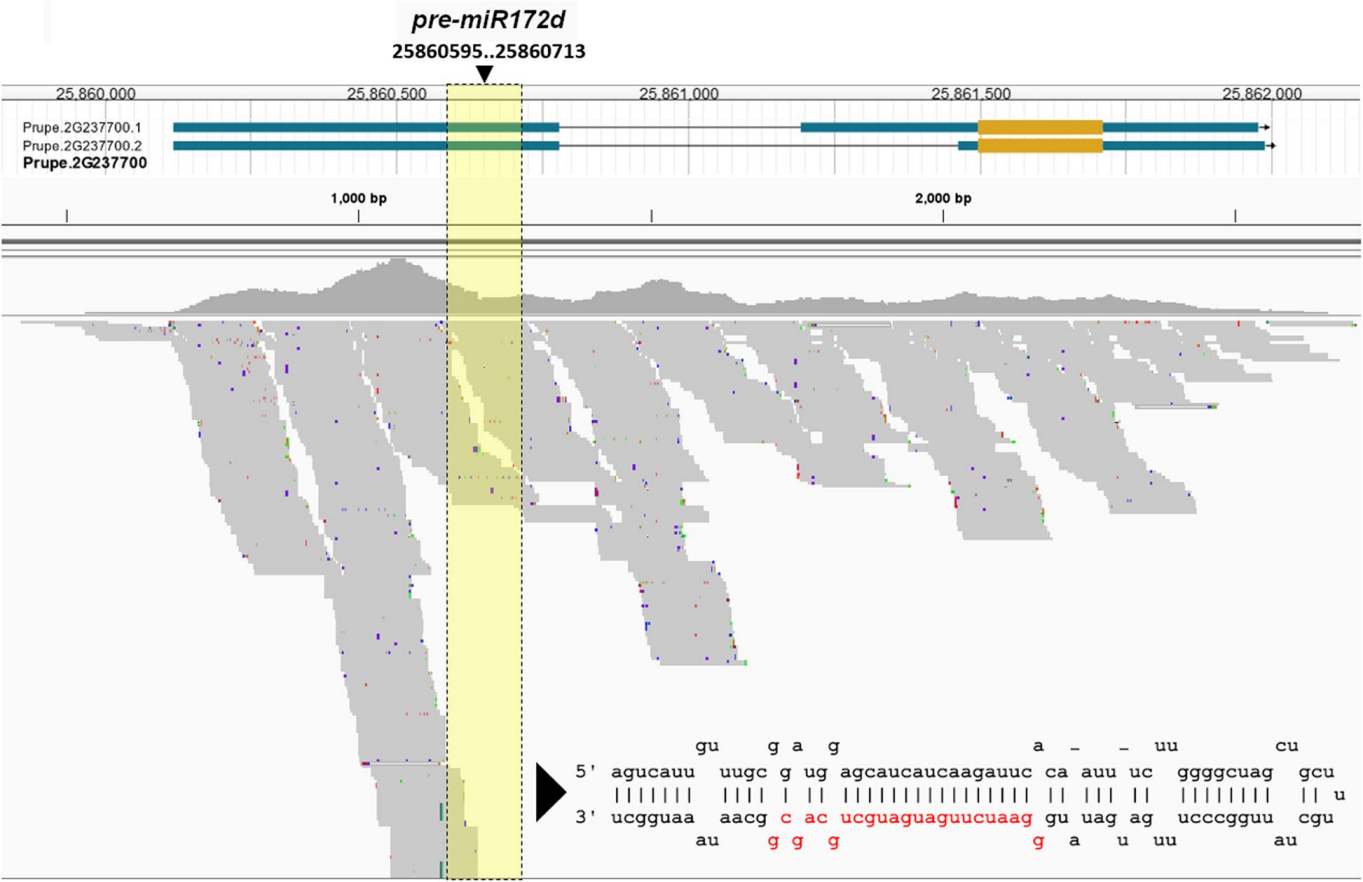
Genomic structure of the micro-RNA *miR172d* encoding transcript *Prupe.2G237700*. The pri-miRNA sequence was identified by mapping transcriptome data from various peach flower tissues (petal, stamen, carpel). The predicted *pre-miR172*d stem-loop hairpin structure is also shown

In the attempt to more precisely delimit the *di* locus position on chr 2, linkage disequilibrium (LD) pattern around the 23 – 26.5 Mb genomic interval enclosing the two associated GWAS signals was inspected in 21 Oriental DF accessions, using about 4K SNPs extracted from whole-genome sequencing (WGS) data [21]. The LD map showed a complex pattern characterized by spaced and relatively short regions with long-range high LD levels, defining an interval of about 2.4 Mb, approximately comprised between SNP1456516 (24,277,038) and SNP1501405 (26,628,544) (Supplementary Figure S1). At least 381 gene models were annotated in this interval, based on a peach reference transcripts v2.1 annotations (Supplementary Table S1). Homology-based searches of genes putatively involved in flower tissues differentiation, growth and/or development allowed the identification of a predicted gene (*Prupe.2G237700*), transcribing a microRNA precursor sequence belonging to the miR172 family, previously classified as *ppe-miR172e* [28] and hereafter named *ppe-miR172d* (as currently annotated on www.mirbase.org, accession: MI0026098). Indeed, an increasing body of knowledge around the role of the euAP2/TOE-miR172 module in the regulation of petaloidy in various species [27] makes *Prupe.2G237700* a prime candidate for the recessive DF trait. Firstly, the sequence of the *miR172d* primary transcript was annotated by mapping RNA-seq reads from peach dormant buds of various stages and carpel tissues. The expressed *pri-miR172d* sequence has no introns, spanning ~2.0 Kb from 25,860,117 to 25,861,987 bp, with the predicted *pre-miR172d* precursor sequence (119 nt) starting at 25,860,595 (Fig. 1).

### Three independent mutant alleles of *Prupe.2G237700* are associated with the recessive double-flower trait

**Fig. 2:**
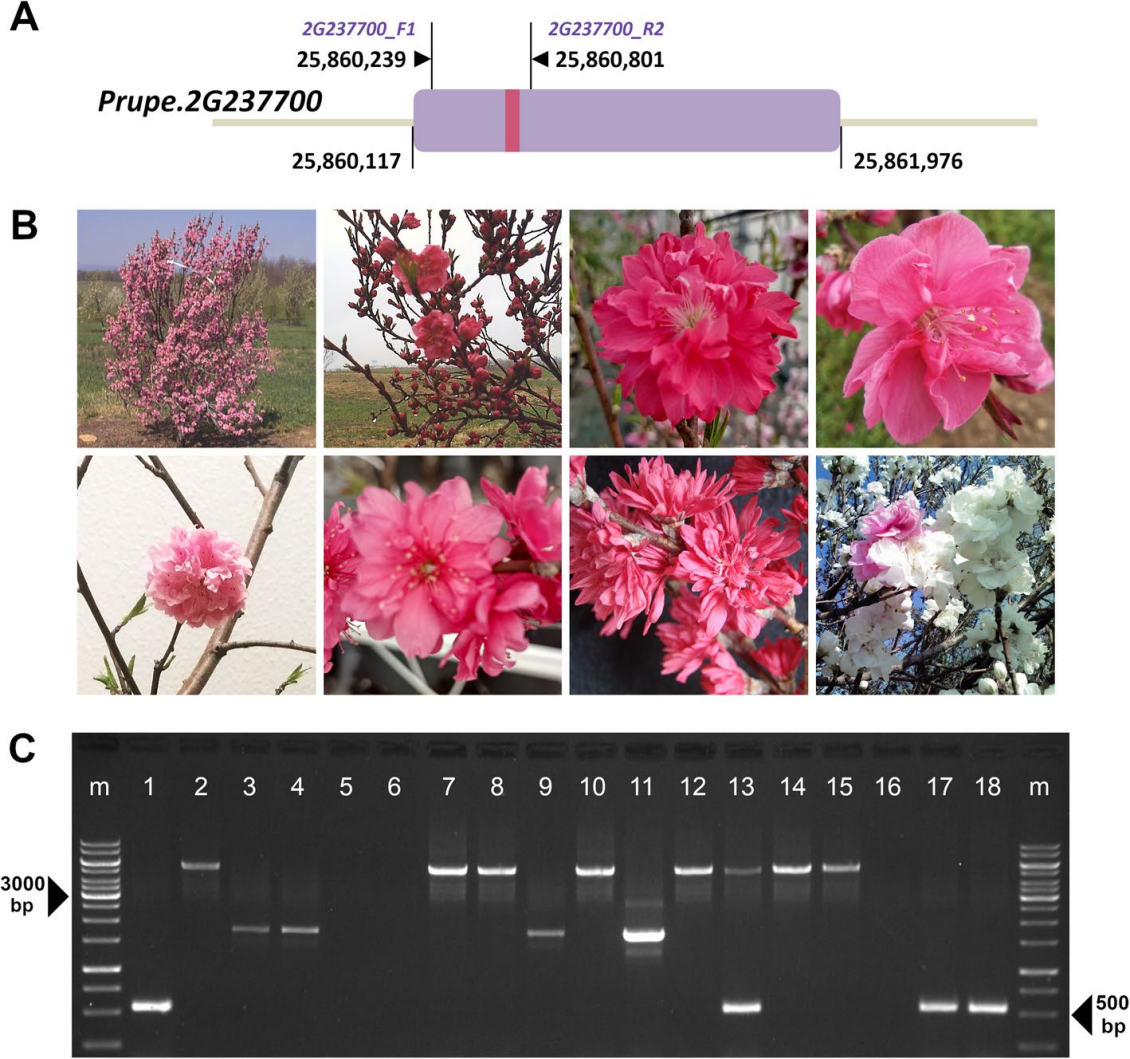
Molecular analysis of sequence variants of the *miR172d* gene. **A** Genomic structure of *Prupe.2G237700* (purple box) and position of *pre-miR172d* encoding sequence (red segment). Arrowheads indicate the positions of the primers used for PCR analysis. **B** Phenotype of double-flower accessions used in this study. Left to right, Top: ‘KV872615’, ‘Compact Pillar’, ‘S7258 cl. IRAN 6:59’, ‘S10321 Hua 5-15’; Bottom: ‘Klara Meyer’, ‘S10322 Hua 5-25’, ‘Kikoumomo D (KV044770)’, ‘Hokimomo *op*’ (trees and flowers photos were provided by C. Dardick and V. Signoret). **C** PCR analysis of the single and double-flower accessions listed in Table 1

**Fig. 3:**
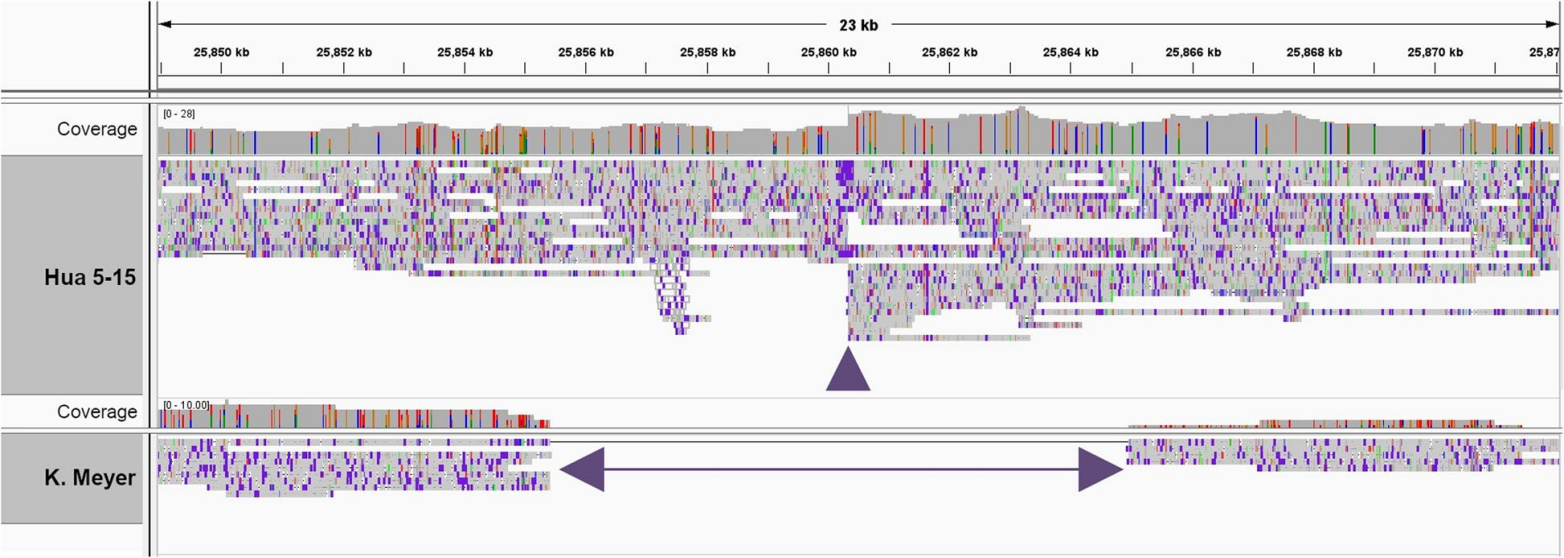
ONT long-sequencing reads alignment at *di* locus in ‘S10321 Hua 5-15’ and ‘Klara Meyer’ accessions. At the top: reads breakpoint due to a ~5.0 Kb LTR insertion (*di*^*1*^) located at position +221 (Pp02:25,860,338) from the *pri-miR172d* transcription initiation site. At the bottom: a ~9.5 Kb deletion (*di*^*Δ*^) from (Pp02:25,855,441 to 25,864,957), encompassing the entire pri-miR172 precursor sequence including its flanking DNA regions in ‘Klara Meyer’

Several primer pairs covering the entire *pri-miR172d* sequence plus ~1 Kb upstream and downstream were designed to analyze allelic polymorphisms in various ornamental materials, including main commercial cultivars, and accessions or selections retrieved from reliable germplasm collections of INRAE-GAFL (France), University of Milan (Italy), USDA-ARS (Kearneysville, West Virginia, and Byron, Georgia) and Clemson University (South Carolina) (the accession list is provided in Table 1). In agreement with the recessive inheritance of the *di* trait, causal variant(s) were assumed to be homozygous in DF genotypes. Interestingly, primers 2G237700_F1 and 2G237700_R2 resulted the most informative, as PCR showed a diverse pattern of amplifications in the various accessions (Fig. 2): in single-flower (SF) accession ‘Crimson Rocket’ the wild-type (*Di*) amplicon of 563 bp (SF ‘Lovell’ peach genome) was observed, as well as in two DF selections ‘KV045590’ and ‘KV021779’; another SF accession, ‘Redleaf Pillar’, showed the 563 bp amplicon alongside a second PCR product of about 5.5 Kb. This PCR product, about 5.0 Kb longer than the wild-type (*Di*) allele, was exclusively obtained in DF accessions ‘Hokimomo op’, KV872615, ‘Compact Pillar’, ‘Peppermint Stick’, S10321 ‘Hua 5-15’, S10322 ‘Hua 5-25’ and ‘Okinawa’. Interestingly, the other four DF accessions (S7258 ‘clone IRAN 6:59’, ‘Kikoumomo D’, ‘G. Biffi fiore doppio’ and ‘Helen Borchers’) showed an amplicon of ~1.7 Kb (suggestive of an insertion of ~1.2 Kb compared to the *Di* allele). Finally, three additional DF accessions showed no amplification (among them ‘Klara Meyer’). Sanger sequencing of the 1.7 Kb amplicon revealed a 1,198 bp insertion without LTR elements (named *di*^*2*^ located at position +297 (Pp02:25,860,414) from the pri-miR172d transcription initiation site (Supplementary File S1). BLAST searching showed no clear similarity with known genes or transposable elements, although identical sequences are spread across several peach chromosomes. Instead, a long-read Oxford Nanopore Technology (ONT) approach was used to sequence the *miR172d* allelic variants in ‘Klara Meyer’ and ‘S10321 Hua 5-15’, as representative of the genotypes respectively yielding no amplification and the 5.5 Kb amplicons with *2G237700_F1* and *2G237700_R2*. Whole-genome coverage was ~10 and ~20x, respectively for ‘Klara Meyer’ and ‘Hua 5-15’, with estimate read length N50 of 32.5 and 21.2 Kb. After aligning to the Peach V2.0 reference genome, two different allelic variants were discovered at the *miR172d* locus: a ~9.5 Kb deletion (Pp02:25,855,441 to Pp02:25,864,957) encompassing the whole miR172 miRNA precursor sequence including its flanking DNA regions, named *di*^*Δ*^; a ~5.0 Kb LTR insertion (hereafter *di*^*1*^), located at position +221 (Pp02:25,860,344) from the *pri-miR172d* transcription initiation site (Fig. 3).

**Table 1:** List of peach ornamental accessions and selections analyzed in this study. Information about the genotype at *di* and *Di2* loci, as well as source of material, origin and basic description of flower phenotypes are also provided. Asterisks indicate the presence of PET mutation at *Di2* locus

#	Accession	Origin and description	Source	Flower type	Genotype
1	Crimson Rocket	complex cross from NJ Pillar and Italian Pillar	USDA-ARS (Kearneysville, West Virginia)	Single	*Di/Di*
2	Hokimomo *op*	Japan, variegated white and pink flowers	USDA-ARS (Kearneysville, West Virginia)	Double	*di*^*1*^*/di*^*1*^
3	S7258 cl. IRAN 6:59	Iran	INRAE-GAFL (Avignon, France)	Double	*di*^*2*^*/di*^*2*^
4	'G. Biffi' Fiore Doppio	unknown, Italy	University of Milan (Milan, Italy)	Double	*di*^*2*^*/di*^*2*^
5	Taoflora pink	Vietnam – breeding	Nursery	Double	*di*^*Δ*^*/di*^*Δ*^
6	Taoflora white	Vietnam – breeding	Nursery	Double	*di*^*Δ*^*/di*^*Δ*^
7	KV872615	from New Jersey Pillar, pink flowers	USDA-ARS (Kearneysville, West Virginia)	Double	*di*^*1*^*/di*^*1*^
8	Compact Pillar	from New Jersey Pillar, red flowers	USDA-ARS (Kearneysville, West Virginia)	Double	*di*^*1*^*/di*^*1*^
9	Helen Borchers	unknown, USA	USDA-ARS (Byron, Georgia)	Double	*di*^*2*^*/di*^*2*^
10	Peppermint Stick cl.	unknown, USA	Clemson University (Clemson, South Carolina)	Double	*di*^*1*^*/di*^*1*^
11	Kikoumomo D (KV044770)	*op* from Kikoumomo (Japan), chrysanthemum red flowers	USDA-ARS (Kearneysville, West Virginia)	Double	*di*^*2*^*/di*^*2*^
12	S10321 Hua 5-15	China	INRAE-GAFL (Avignon, France)	Double	*di*^*1*^*/di*^*1*^
13	Redleaf Pillar	NJ Pillar (germplasm) x Italian Pillar (germplasm)	USDA-ARS (Kearneysville, West Virginia)	Single	*Di/di*^*1*^
14	S10322 Hua 5-25	China	INRAE-GAFL (Avignon, France)	Double	*di*^*1*^*/di*^*1*^
15	Okinawa	Japan	University of Milan (Milan, Italy)	Double	*di*^*1*^*/di*^*1*^
16	Klara Meyer	Germany, red flowers, semi-dwarf	Kaneppele nursery (Italy)	Double	*di*^*Δ*^*/di*^*Δ*^
17	KV045590	F_2_ from Italian Pillar, white flowers, weeping	USDA-ARS (Kearneysville, West Virginia)	Double	Di/Di*
18	KV021779	From Italian Pillar, rose flowers	USDA-ARS (Kearneysville, West Virginia)	Double	Di/Di*

The *di*^*1*^ insertion showed homology with a putative FRS5-LIKE gene of the FARS (FAR-RED IMPAIRED RESPONSE1 RELATED SEQUENCE) transcription factors family derived from Mutator-like element (MULE) transposases [29]. Validation of the three allelic variants of the *di* locus was performed through PCR analyses. Specific primer combinations confirmed the presence of the homozygous *di*^*1*^ insertion found in ‘Hua 5-15’ also in other six DF accessions: ‘Houki Momo op’, ‘KV872615’, ‘Compact Pillar’, ‘Peppermint Stick cl.’, ‘Hua 5-25’ and ‘Okinawa’ (Supplementary Figure S2); consistent with the recessive inheritance of the *di* trait, the *di*^*1*^ allele was amplified in the heterozygous SF accession ‘Redleaf Pillar’. Finally, the two remaining DF selections ‘KV045590’ and ‘KV021779’ showing the wild-type *Di/Di* genotype (i.e. 563 bp amplicon) both resulted heterozygous (*Di2/di2*) for the PETALOSA variant (underlying the dominant inherited *Di2* allele) [24] (Table 1). In addition to the panel of ornamental cultivars, the absence (in homozygosis) of any of the three identified *di* variant(s) in single-flower accessions was further confirmed in a genetically diversified panel of 151 accessions from the *PeachRefPop* collection [30] (data not shown).

**Fig. 4:**
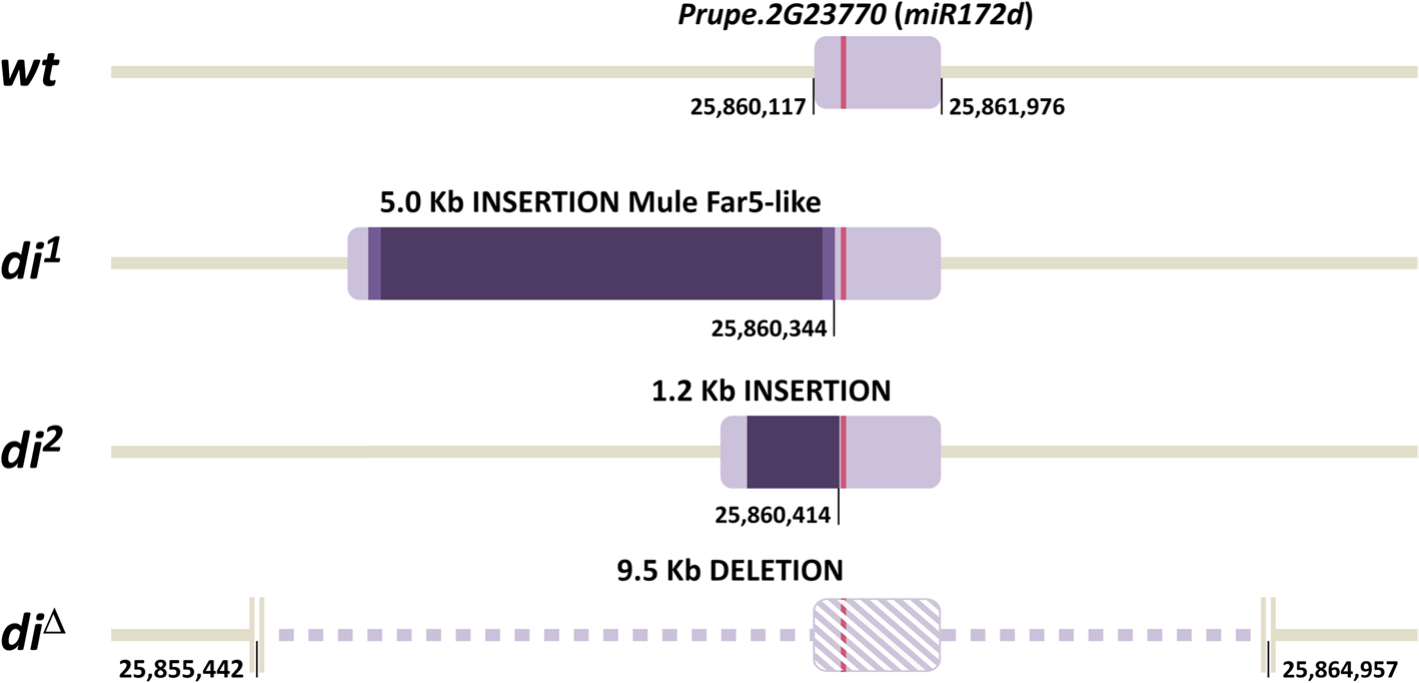
Structural variants identified at the gene encoding micro-RNA *miR172d*, *Prupe.2G237700*. The wt genomic structure of *Prupe.2G237700* is represented by a light lilac box and the red segment indicates the position of the *pre-miR172d* encoding sequence. Insertions in alleles *di*^*1*^ and *di*^*2*^ are represented by a dark purple box, with LTR regions marked in lighter purple in *di*^*1*^. The genomic region deleted in *di*^*Δ*^ is indicated by the dotted/shaded region

Thus, at least three distinct mechanisms acting on the *miR172d* gene underpin the recessive double flower trait in peach: *di*^*1*^ (a ~5.0 Kb insertion), *di*^*2*^ (a ~1.2 Kb insertion) and *di*^*Δ*^ (a ~9.5 Kb deletion) (Fig. 4).

### *Pre-miR172d* expression is abolished in homozygous *di*^*1*^, *di*^*2*^ and *di*^*Δ*^ genotypes

**Fig. 5:**
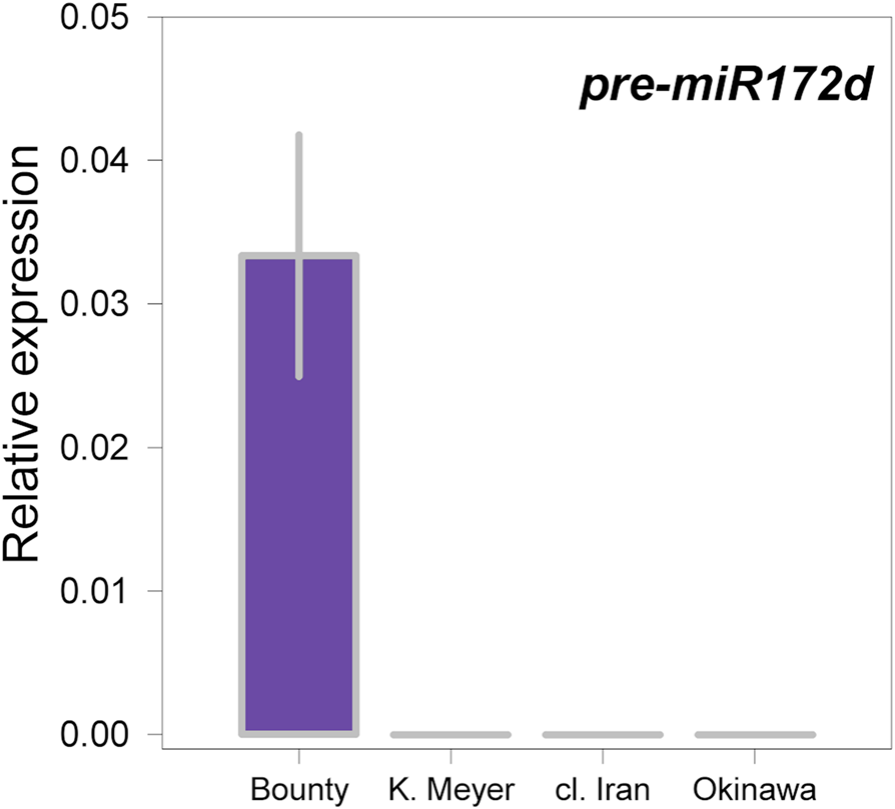
Expression pattern of *pre-miR172d* in pooled flower bud tissues from Baggiolini stage B (flower bud swelling) to C1 (flower buds apparent) from ‘Bounty’ (single-flower), ‘Klara Meyer’ (double-flower, *di*^*Δ*^), ‘S7258 cl. IRAN 6:59’ (double-flower, *di*^*2*^) and ‘Okinawa’ (double-flower, *di*^*1*^) genotypes. *Actin* (*Prupe.6G163400*) was used as a reference to normalize expression data

The expression profile of *pre-miR172d* was investigated using qRT-PCR in pooled bud flower tissues from two developmental stages and three *di* variants: ‘Okinawa’ (*di*^*1*^*/di*^*1*^), ‘clone IRAN 6:59’ (*di*^*2*^*/di*^*2*^) and ‘Klara Meyer’ (*di*^*Δ*^*/di*^*Δ*^). The *pre-miR172d* was only expressed in the SF control accession ‘Bounty’ (*Di/Di*), being undetectable in all the three DF accessions (Fig. 5). These findings were clearly consistent with the complete deletion of *miR172d* sequence in the *di*^*Δ*^ allele; in case of *di*^*1*^ and *di*^*2*^ alleles, transposon-induced gene silencing mechanisms could be the most probable explanation, although the exact mechanism(s) leading to their miss-expression should be more specifically addressed.

### Distribution of allelic variants in DF varieties of germplasm collections

The availability of phenotypic and genotypic information on peach ornamental accessions across important Chinese collections, allowed exploring the allelic assortment at both *di* and *Di2* loci. The presence of allelic variants (and co-segregation with flower phenotype) was assessed through a sequencing-based method (Supplementary Figure S3 and Materials and Methods section) using public WGS data (retrieved from NCBI SRA database) of 41 peach accessions with clear flower trait (double or semi-double) and sufficient genome coverage (i.e. > 10x), and several SF accessions as control. Among the DF accessions, 21 were homozygous *di*^*1*^/*di*^*1*^ (including the dwarf DF ‘Bonanza peach’, probably erroneously indicated as SF), 7 homozygous *di*^*2*^/*di*^*2*^ and ‘Ju Hua Tao’ carried the *di*^*1*^/*di*^*2*^ allelic combination (Supplementary Table S2); in this last case, the concomitant presence of *di*^*1*^ and *di*^*2*^ alleles suggests that both mutations are functionally equivalent; surprisingly, the *di*^*Δ*^ variant seems to be absent in Chinese germplasm collections, suggesting the hypothesis that this allele originated after the arrival of peach in Europe (tracing back to the Greek-Roman era). Apart from the *di* locus, 7 DF accessions resulted heterozygous for the PET-Di2 variant (Supplementary Table S2). Interestingly the DF accession ‘Hua Yu Lu’ was homozygous for *Di* and *di2* alleles but showing a heterozygous SNP within the core miR172 recognition sequence at the three-prime end portion of the coding sequence of the *PET* gene (Supplementary Figure S4).

## Discussion

The pivotal role of euAP2/miR172 regulatory module is a key component of the floral C-function [7]. In flowers expressing a miR172-resistant version of AP2/TOE or with reduced miR172 activity, the stamens were partially (or completely) converted into petals [14, 27]. In tomato, *miR172c* and *miR172d* seem the most abundant forms in developing flowers [31], as also observed in peach [28]. CRISPR-Cas9-editing of tomato *miR172c *and *miR172d* showed that hypomorphic and loss-of-function alleles of *mir172d* were associated with the conversion of petals and stamens to sepaloids, suggesting a dose-dependent regulation of floral organ identity and number [31]. A very recent association study on 417 peach accessions from the Peach Germplasm Repository of Shandong (Agricultural University, China) also suggests the presence of two independent insertion events (named *Hap2* and *Hap3*) within the promoter of *Prupe.2G237700* [32] in DF accessions. Sequence alignment and insertion position both confirm the identity of *Hap2* with the *di*^*2*^ allele. Also, the high degree of similarity (97.6%) suggests a correspondence of the *Hap3* insertion with the *di*^*1*^ allele, despite a 500 bp longer sequence (5.5 vs 5.0 Kb, respectively). The different insertion size between the two alleles may arise from the assembly methods used for obtaining the consensus sequence (i.e. ONT long reads for *di*^*1*^ , Illumina short-reads for *Hap3*). A length of 4,992bp for the *di*^*1*^ insertion seems to be consistent with the amplicon sizes obtained by PCR (Fig. 2). Moreover, homology searches resulted in no exact matches in the Peach reference genome, in contrast to *Hap3*, showing 100% identity with an LTR sequence on chr 4 (18,058,479 - 18,063,978). Furthermore, as demonstrated by the annotation of *miR172d* primary transcript, both insertions fall within the expressed *pri-miR172d* sequence and not in its promoter region (Figs. 1 and 4), and their presence appears to abolish its expression as much as the putatively *null di*^*Δ*^ allele (Fig 5).

Apart from the molecular aspects, the *di*^*1*^, *di*^*2*^ and *di*^*Δ*^ allelic variants identified in the present work add valuable information on the genetics of ornamental cultivars, also allowing an evaluation of the breeding history of the varieties used in this study. ‘Houki Momo’ is a seed-propagated ancient ornamental peach from the Edo Era (1700 – 1900) in Japan, characterized by a fastigiated habitus and white-pink double flowers [33]. ‘Houki Momo’ was either the parent or possibly just renamed 'NJ Pillar' (C. Dardick, personal communication). 'Houki Momo‘ and ‘NJ Pillar’ were also the donor of DF trait of several commercial ornamental cultivars, such as the 'Terute’ (and ‘Terute momo’ and ‘Teruteshiro’, from F_2_ crosses with ‘Akashidare’ and ‘Zhu Fen Chui Zhi’) and the ‘Corinthian’ series of DF cultivars [34, 35], as well as some selections of USDA-ARS (such as ‘Compact Pillar’). Noteworthy, 'NJ Pillar' was one of the cross-parent of the segregating progeny used for the early mapping of *di* locus [17]. Moreover, 'NJ Pillar' and ‘Italian Pillar’ were also the source of fastigiated and upright growth habits in peach breeding [36], although the latter has single flowers. ‘Peppermint Stick’ (also known as ‘Candy stick’ or ‘Variegated’) has white (or pink) flowers with red stripes and was probably selected from ‘Versicolor’ cultivars around the mid-XIX century, although introduced to the US market only in 1939 by Clarke Nursery (San Jose, California) [37] and still commercially available. The recessive inheritance of DF trait in progenies issued from this cultivar was early reported by Lammerts (1945). ‘Okinawa’ is a low-chill rootstock characterized by white and semi-double flowers, originated from a seed lot imported by the Florida Agricultural Experiment station from the Ryukyu Islands in 1953 [38]. PCR also validated the homozygous *di*^*Δ*^ deletion in ‘Klara Meyer’ and the two DF accessions yielding no amplicons with *di_miR172F*/*di_miR172R*: ‘Taoflora Pink’ and ‘Taoflora White’ (Supplementary Figure S2). 'Klara Meyer' is an old ornamental peach dating back to 1860 from France [39] (probably named in honor of the homonymous German actress) and bearing dark pink flowers with 80 - 90 petals [40]; this cultivar was introduced in the United States at the end of XIX century (1891) by Spath nursery. By contrast, the ‘Taoflora’ series was relatively recent, released in 2007 by the breeder N’Guyen Dinh Mao (Vietnam), although the breeding source of *di*^*Δ*^ is unknown. Regarding the *di*^*2*^ variant, its breeding origin has been more elusive. Of the three *di*^*2*^ genotypes, the USDA-ARS selection ‘Kikumomo D’ (‘KV044770’) was obtained from an open-pollination of the Japanese ‘Kikumomo’ (probably also known as ‘Ju Tao’) showing the same chrysanthemum-type (non-showy) flowers. Progenies from ‘Kikumomo’ showed a recessive inheritance of DF trait [41]. 'Helen Borchers' was one of the oldest US commercial ornamental peaches released before 1939 by Clarke Nursery [16], bearing pink flowers with 30 – 40 petals, 10 sepals and multiple pistils [40]. Segregation of flower doubleness in F_2_ progenies from a F_1_ seedling derived from ‘Helen Borchers’ further confirmed the recessive inheritance of its *di*^*2*^ allele [16]. Our analysis clearly shows that the flower doubleness of ‘Helen Borchers’ is not ascribable to a *di*^*Δ*^ allele derived from ‘Klara Meyer’, that was previously deemed as its parent [37].

Apart from the peach ornamental accessions available in European and US collections, wider germplasm pools are maintained in Far-East countries, which boast a centennial tradition in the conservation, selection and breeding of ornamental cultivars. A major difficulty in charting the breeding history of the *di* locus arises from the uncertain identity and classification of many cultivars, probably as a consequence of the long lasting material exchanges via seeds or bud-sticks, particularly across China, Japan, and more recently Western countries. Despite this, phylogenetic and population structure studies have consistently shown an evident differentiation of the ornamental peach and hybrids clusters (from *P. davidiana*, such as ‘Bai Hua Shan Bi Tao’ and ‘Fen Hua Shan Bi Tao’) from the ‘edible’ peach groups of landraces and improved cultivars [42–44]. Also, a certain degree of subpopulation differentiation has been reported within the ornamental peach cluster [35, 45], although the genetic relationships among the subgroups mainly reflect the growth habits (i.e. dwarf, fastigiated, weeping, upright or standard) rather than a (putative) different origin of DF alleles. Furthermore, different mutations from those described could also have arisen in different collections, and propagated locally. An example could be ‘Hua Yu Lu’ in which the reported DF phenotype could be due to an heterozygous SNP within the miR172 recognition sequence of the *PET* gene (Supplementary Figure S4). While the effective presence of this polymorphism should be further validated, the effects of single nucleotide mutations altering the miR172 seed region in *PETALOSA* gene orthologs have already been proven to induce the development of extra-numerary petals in gene-edited tobacco plants [27]. Other than the induction of flower supernumerary organs, *di* allelic variants cannot explain the range of differences in the number of extra-petals found in recessive DF accessions. In case of the dominant PET-Di2 mutation, an allele–dosage effect on average petal number explained most of the variability, with an increase of supernumerary petals in homozygous (*Di2/Di2*) versus heterozygous (*Di2/di2*) individuals [24], but the recessive nature of *di* mutations excludes such an effect (i.e. heterozygous *Di/di* individuals have single-flowers). While the de-regulation of PETALOSA genes has been indicated as responsible for the formation of extra-numerary flower structures in a variety of species, it is likely that additional layers of interaction between different euAP2 and/or MADS-box genes are in place to regulate flower organ formation. In a *miR172d*-deficient background, genotype-specific differences in the expression pattern of other homeotic genes could affect both meristem size and the conversion of stamens into petaloid structures. The existence of different interacting factors in determining petal number in *di/di* backgrounds had already been put forward by Lammerts (1945), and it has to be taken into account that miR172 is a key regulator of all euAP2 genes, and it is therefore expected that in *miR172d*-deficient individuals the activity of additional euAP2 could be prolonged. Transcripts for two euAP2 genes (*Prupe.6G231700* and *Prupe.6G091100*) other than that for PETALOSA (*Prupe.6G242400*) were already reported in developing peach buds [24], but their precise tissue specificity and role in flower development are not known. Further work would be necessary to dissect their function and the effects of a possible de-regulation of the transcripts of these genes in the floral meristem a *di/di* background, taking also into account that environmental cues could also play a role: RcAP2 (a non-PETALOSA euAP2 gene sharing high similarity with *Prupe.6G231700*) has been suggested to regulate the conversion of stamens into petals in DF roses grown in different temperature conditions, with a variation in petal number of to up to three-fold [46]. Furthermore, in peach, a strong correlation has been observed between blooming date and either petal or sepal number in different segregating progenies, suggesting a link between flower morphology and phenology [30, 47]. Therefore, also in light of the changing climate conditions in many parts of the world, different genetic, environmental and/or G x E factors will have to be taken into consideration not only for crop species but also for ornamentals: to these ends, the information and characterized mutants already obtained in peach will be extremely valuable for future studies.

## Conclusions

The identification of three independent mutations associated with the *di* locus responsible for the recessive double-flower phenotype unequivocally demonstrated the essential function of miR172 for floral identity and patterning in peach. These findings reinforce the evidences around the pivotal role of the miR172/euAP2 module and associated genetic variants in the creation of diverse flower forms and shapes, as previously advanced by the discovery of miR172-insensitive PETALOSA alleles. The allelic spectrum at the *di* (miR172d) and *Di2* (*PETALOSA* gene) loci explains almost completely the appearance of DF in peach. This information may be translated to other species and pave the way to an understanding of the formation of DF in different ornamentals.

## Methods

### Plant materials

Leaf samples and photos of the 18 peach accessions used in this study were kindly provided by institution from various countries or directly acquired from nurseries, as specified in Table 1: accessions ‘Crimson Rocket’, ‘Hokimomo op’, KV045590, KV021779, Redleaf Pillar, Kikoumomo D (KV044770), KV872615 and Compact Pillar by C. Dardick (USDA-ARS, Appalachian Fruit Research Station, Kearneysville, West Virginia); ‘Helen Borchers’ by Chen Chunxian (USDA-ARS, Southeastern Fruit and Tree Nut Research Laboratory, Byron, Georgia; ‘Peppermint Stick redleaves clone’ by K. Gasic (Clemson University, Clemson, South Carolina); ‘S10321 Hua 5-15’, ‘S10322 Hua 5-25’ and the orginal source of ‘S7258 clone IRAN 6:59’ by B. Quilot (INRAE, Unit Genetics and Breeding of Fruit and Vegetables, Avignone, France). Commercial cultivars ‘Taoflora pink’ and ‘Taoflora white’ were provided by private nurseries. Trees of the accessions ‘Klara Meyer’ (from Kaneppele nursery, Trentino-Alto Adige, Italy), ‘S7258 clone IRAN 6:59’, ‘G. Biffi fiore doppio’ and ‘Okinawa’, as well as additional 151 accessions from the *PeachRefPop* collection were maintained at ASTRA-M. Neri farm ‘La Brusca’ (Imola, Italy). Accessions can be divided into either single or double flowers, the last including both double and semi-double types.

### MinION library preparation, sequencing and analysis

High molecular weight (HMW) DNA was extracted following the protocol described by Li *et al*. [48]. The integrity and purity of the extracted DNA were evaluated in a 0.5% agarose gel electrophoresis run and with Thermo Scientific Multiskan GO (Thermo Fischer Scientific), respectively. DNA was quantified using the Invitrogen Qubit fluorimeter (Thermo Fisher Scientific, Massachusetts). Library preparation for amplicon sequencing was achieved using the 1D Genomic DNA by ligation protocol (SQK-LSK109; ONT, Oxford, United Kingdom), according to the manufacturer’s instructions. Briefly, each sequencing library was prepared using 1.5 μg of high molecular weight genomic DNA extracted from nuclei. Sequencing was performed on a R9.4.1 flow-cell (Oxford Nanopore Technologies, ONT, United Kingdom) on a MinION Mk1b device. Each sample was run individually in a different flow cell. The basecalling of the ONT long reads was performed by Guppy v. 5.0.13 within the MK1C device. Reads were aligned against the peach V2.0 reference genome [49] using GraphMap Aligner [50], converted into bam files using SAMtools software [51]. True alignment was considered for both positive and negative strands following the GraphMap parameters for correct alignment. Each read was assigned with a quality indicator (mapQ), and those reads with a quality score ≥40 were accepted for inferences, while reads with quality rate ≤40 were not considered as a true alignment. IGV software [52] was used for visual exploration of sequencing data and retrieval of informative reads.

### DNA extraction and genotyping

Genomic structure of the micro-RNA gene *miR172d* encoding transcript *Prupe.2G237700* was identified by mapping RNAseq reads from various flower tissue libraries (carpel, petal, stamen, PRJNA493230) against peach V2.0 genome reference using STAR [53]. Genomic DNA was extracted from 200 μg of leaf tissue using a CTAB modified method [54] and 20 ng for each sample were used in PCR reactions using GoTaq Long PCR Master Mix (Promega) in a total volume of 10 ul and the appropriate primer combinations (Supplementary File S1), 5’-3’ strand: *2G237700_F1* (GTTTTACCATGTGGTCCCTGGG) and *2G237700_R2* (GACCAGTATCTAAATGTTTTACCTG) as *pre-miR172* specific primers; *diDEL_A_For* (CAACATTGGATTACACACACTTC), *diDEL_B_Rev* (TGTCCAGTATAAACTGTAGTGGC), *diDELwt_C_For* (CGCTGATATTTGAAGGGTTTCAC) and *diDELwt_D_Rev* (TGTCAGAAACTTGATTCCAAGAC) specifics for *di*^*Δ*^ variant and wild-type control; *2G237700_F1* and *2G237700_di1_Rev* (ACTCATCAGCCACATCAGATGG) for *di*^*1*^ variant; *2G237700_di2_For* (AGGACTATTTAACCCAGTCCAG) and *2G237700_R1* (TCATGATATCATCACGCCCATG) for *di*^*2*^ variant. PCR cycles were as follow: initial denaturation at 94° C for 30 sec., annealing at 58° C for 30 sec., extension at 65° for 4 min. (32 cycles) and final extension at 72° C for 5 min. The *PETALOSA* deletion variant at the *Di2* locus was genotyped using primers reported in Gattolin *et al.* [24].

### RNA Isolation and RT-qPCR analysis

Total RNA was collected from pooled flower bud tissues from Baggiolini stage B (flower bud swelling) to C1 (flower buds apparent). RNA was treated with DNase I (Takara) and extracted using TRIzol (Thermo Fisher Scientific) followed by phenol extraction; 1 μg of total RNA was reverse-transcribed using random hexamers and SuperScript III RT (Thermo Fisher Scientific). According to Zhu et al. (2012), specific primers for pre-miR172d amplify a 90 bp region including mature *miR172d*, located 84 nt from the 5’-prime end of the precursor sequence (Supplementary File S1): *pre-miR172d_For* (AGCATCATCAAGATTCACAATTTC) and *pre-miR172d_Rev* (TGCCGCTGCAGCATCATCAAGA). Real-time PCR reaction were carried out using Power SYBR Green qPCR Supermix (Thermo Fisher Scientific). Each reaction was performed in triplicate on QuantStudio 3 Real-Time PCR Systems (Thermo Fisher, United Kingdom) using the following cycling conditions: 95°C for 10 min, 40 cycles at 95°C for 20 s, 56°C for 25 s, and 72°C for 30 s. Each thermal cycle was followed by a melting curve stage, with temperatures ranging from 60°C to 95°C. *Pre-miR172d* relative expression was calculated using ∆C_t_ method [55], using *Actin* (*Prupe.6G163400*) as reference gene [56]. RT-PCR products were run on 2.0% agarose gels with Midori staining and visualized under UV light. Three to four biological pool replicates were analyzed for each sample.

### Sequence-based genotyping

For sequencing-based method, Illumina genomic resequencing data of peach and wild relatives accessions were retrieved from the SRA database of NCBI (Supplementary Table S2). The *pri-miR172d* wild-type genomic regions adjacent to the insertion/deletion breakpoints were blasted against cleaned reads of each accession. Variant(s) were deemed to occur when truncated reads were identified in wild-type sequences, and, as a control, overlapped reads were detected using the specific polymorphic allele.

## Supplementary Information

**Additional file 1: Supplementary Figure S1.** Pattern of Linkage disequilibrium decay around *di* locus (chromosome 2) estimated from whole-genome sequencing data retrieved from Meng et al. (2019) [21].

**Additional file 2: Supplementary Figure S2.** PCR-based identification of *di*^*1*^ and *di*^*Δ*^ alleles at *di* locus and primers positions. Samples 1 – 7: *di*^*1*^ allele specific genotyping in ‘Hokimomo *op*’, ‘KV872615’, ‘Compact Pillar’, ‘Peppermint Stick cl.’, ‘Redleaf Pillar’, ‘S10322 Hua 5-25’ and ‘Okinawa’ accessions with primers *2G237700_F1* and *2G237700_di1_Rev*; samples 8 – 10: *di*^*Δ*^ allele specific genotyping in ‘Taoflora pink’, ‘Taoflora white’ and ‘Klara Meyer’ accessions with primers *diDEL_A_For* and *diDELwt_D_Rev*.

**Additional file 3: Supplementary Figure S3.** Allele mining at *di* and *Di2* loci in peach germplasm using a sequence-based method. Exemplificative outputs were provided for homozygous *di*^1^/*di*^*1*^ and *di*^2^/*di*^*2*^ genotypes (‘Sa Hong Long Zhu Tao’ and ‘Hong Hua Bi Tao’), and a heterozygous *di*^1^/*di*^*2*^ (‘Ju Hua Tao’). Arrows indicate the position of *di*^1^ (left) and *di*^2^ (right) insertions.

**Additional file 4: Supplementary Figure S4.** Single nucleotide polymorphism detected within the miR172 seed region of *PETALOSA* gene (*Prupe.6G242400*) at the *Di2* locus in the double-flower ‘Hua Yu Lu’ accessions. The *PET* deletion variant near the C terminus (Pp06:24,074,355 - 24,075,350) is also indicated.

**Additional file 5: Supplementary Figure S5.** Picture of the agarose gel electrophoresis of PCR products used in Fig. 2.

**Additional file 6: Supplementary Figure S6.** Picture of the agarose gel electrophoresis of PCR products used in Fig. S2.

**Additional file 7: Supplementary File S1.** DNA sequence of the *di*^*Δ*^*, di*^*1*^ and *di*^*2*^ variants. *Prupe.2G237700* is highlighted in green with the boxed pre-miR172 sequence. The deletion is indicated in *di*^*Δ*^ with strikethrough text while the insertions in *di*^*1*^ and *di*^*2*^ are highlighted in orange and yellow, respectively. Primers used for genotyping are marked as bold, underlined sequences. Primers used for RT-qPCR analysis are shown on a portion of the *pre-miR172d* sequence.

**Additional file 8: Supplementary Table S1.** Gene models were annotated in interval between SNP1456516 (24,277,038) and SNP1501405 (26,628,544), based on a peach reference transcripts v2.1 annotations. Best homology hit and percentage of identity to Arabidopsis gene models are indicated.

**Additional file 9: Supplementary Table S2.** Analysis of allelic variant on WGS data retrieved from NCBI SRA database of 41 peach accessions. The presence of *di*^*1*^/*di*^*1*^, *di*^*2*^/*di*^*2*^ and *di*^*1*^/*di*^*2*^ genotypes or of *PET* alleles in *Prupe.6G242400* was assessed. References to phenotype information about double-flower and other ornamental traits are also indicated. Accessions with unclear genotype at either *di* or *Di2* loci were also included.

## Data Availability

The sequences generated during the current study are available in the Sequence Read Archive (SRA) repository under accession code PRJNA814062
