# Supplementary figures and images for: Less is more: natural variation disrupting a miR172 gene at the di locus underlies the recessive double-flower trait in peach (P. persica L. Batsch)
Source: BMC Plant Biol. 2022 Jul 4;22:318. doi: 10.1186/s12870-022-03691-w (PMC9252053; doi:10.1186/s12870-022-03691-w)

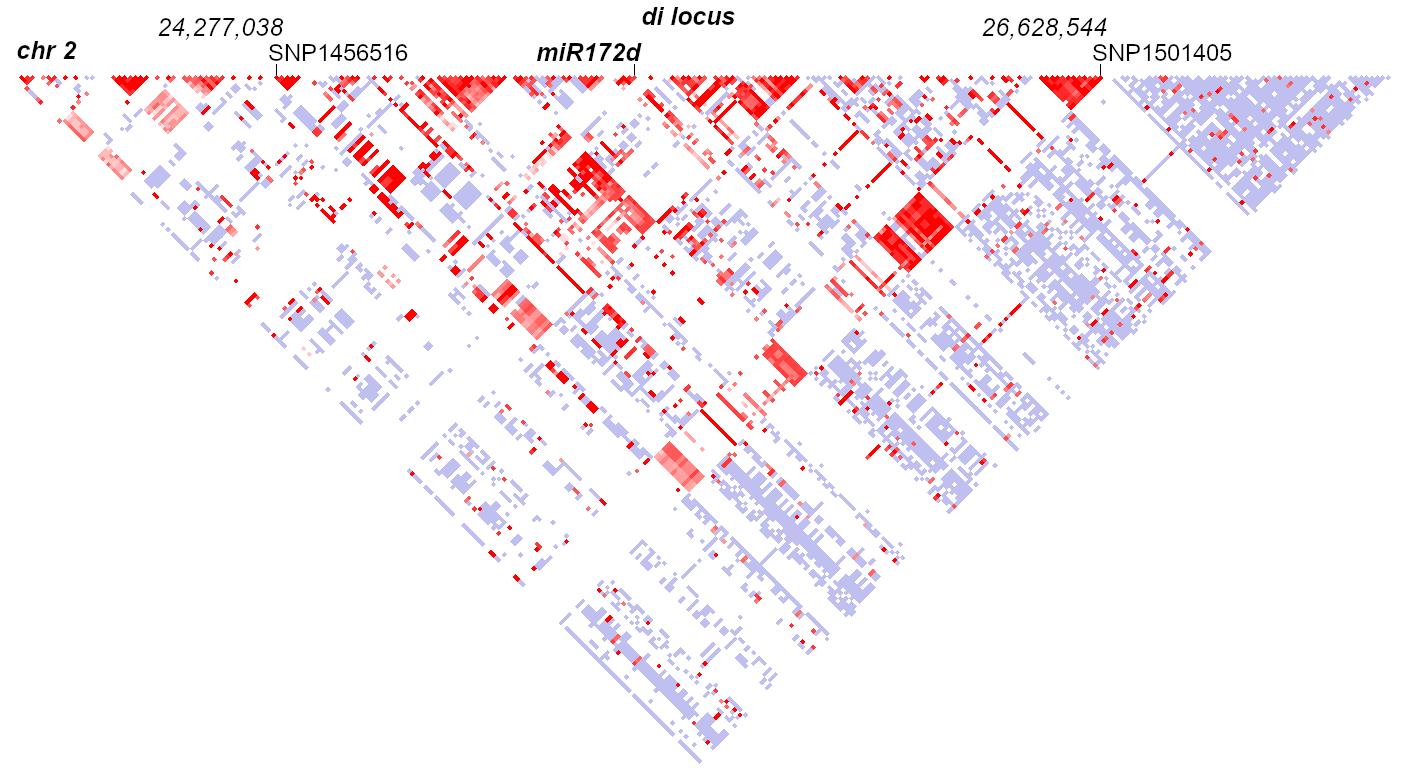

Supplement: Supplementary file 1 — Additional file 1: Supplementary Figure S1. Pattern of Linkage disequilibrium decay around di locus (chromosome 2) estimated from whole-genome sequencing data retrieved from Meng et al. (2019) [21]. [file 12870_2022_3691_MOESM1_ESM.png]

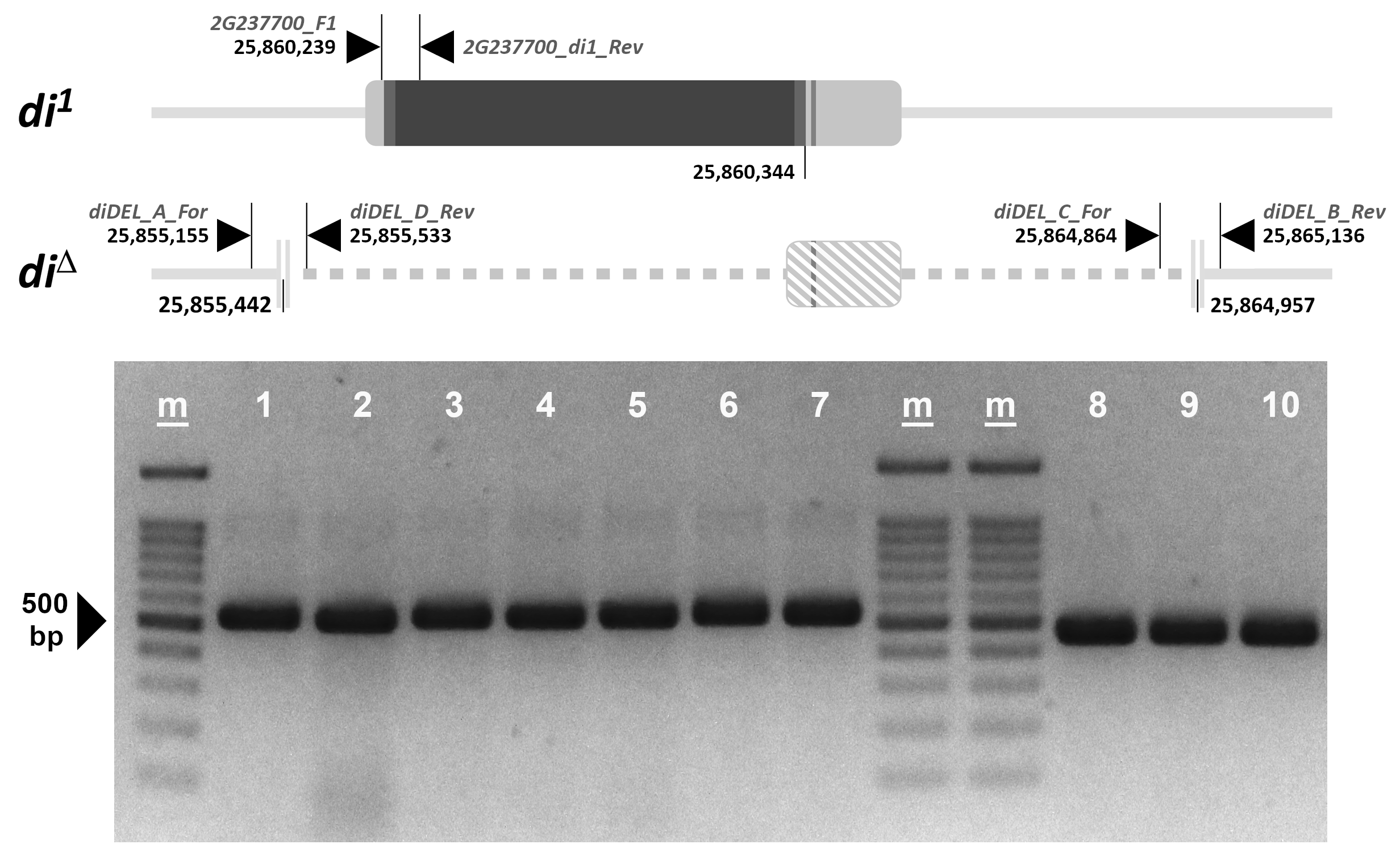

Supplement: Supplementary file 2 — Additional file 2: Supplementary Figure S2. PCR-based identification of di1 and diΔ alleles at di locus and primers positions. Samples 1 – 7: di1 allele specific genotyping in ‘Hokimomo op’, ‘KV872615’, ‘Compact Pillar’, ‘Peppermint Stick cl.’, ‘Redleaf Pillar’, ‘S10322 Hua 5-25’ and ‘Okinawa’ accessions with primers 2G237700_F1 and 2G237700_di1_Rev; samples 8 – 10: diΔ allele specific genotyping in ‘Taoflora pink’, ‘Taoflora white’ and ‘Klara Meyer’ accessions with primers diDEL_A_For and diDELwt_D_Rev. [file 12870_2022_3691_MOESM2_ESM.png]

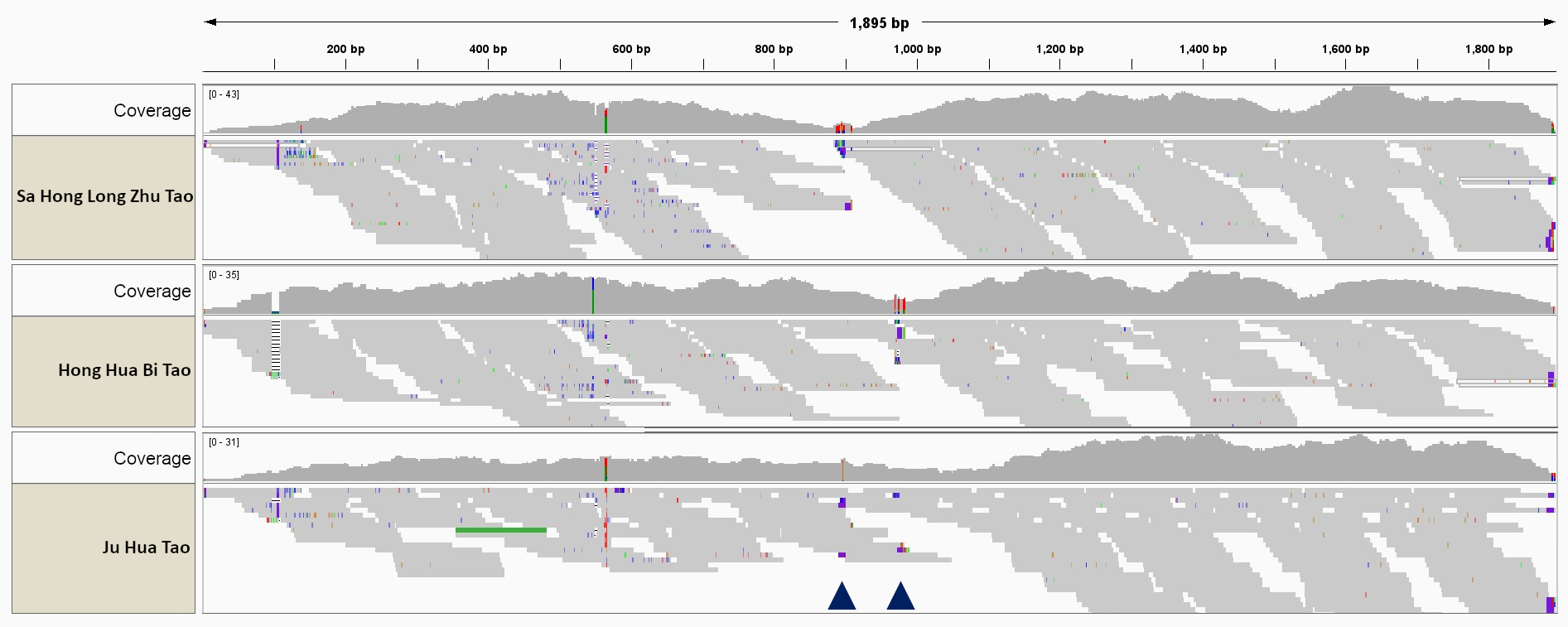

Supplement: Supplementary file 3 — Additional file 3: Supplementary Figure S3. Allele mining at di and Di2 loci in peach germplasm using a sequence-based method. Exemplificative outputs were provided for homozygous di1/di1 and di2/di2 genotypes (‘Sa Hong Long Zhu Tao’ and ‘Hong Hua Bi Tao’), and a heterozygous di1/di2 (‘Ju Hua Tao’). Arrows indicate the position of di1 (left) and di2 (right) insertions. [file 12870_2022_3691_MOESM3_ESM.png]

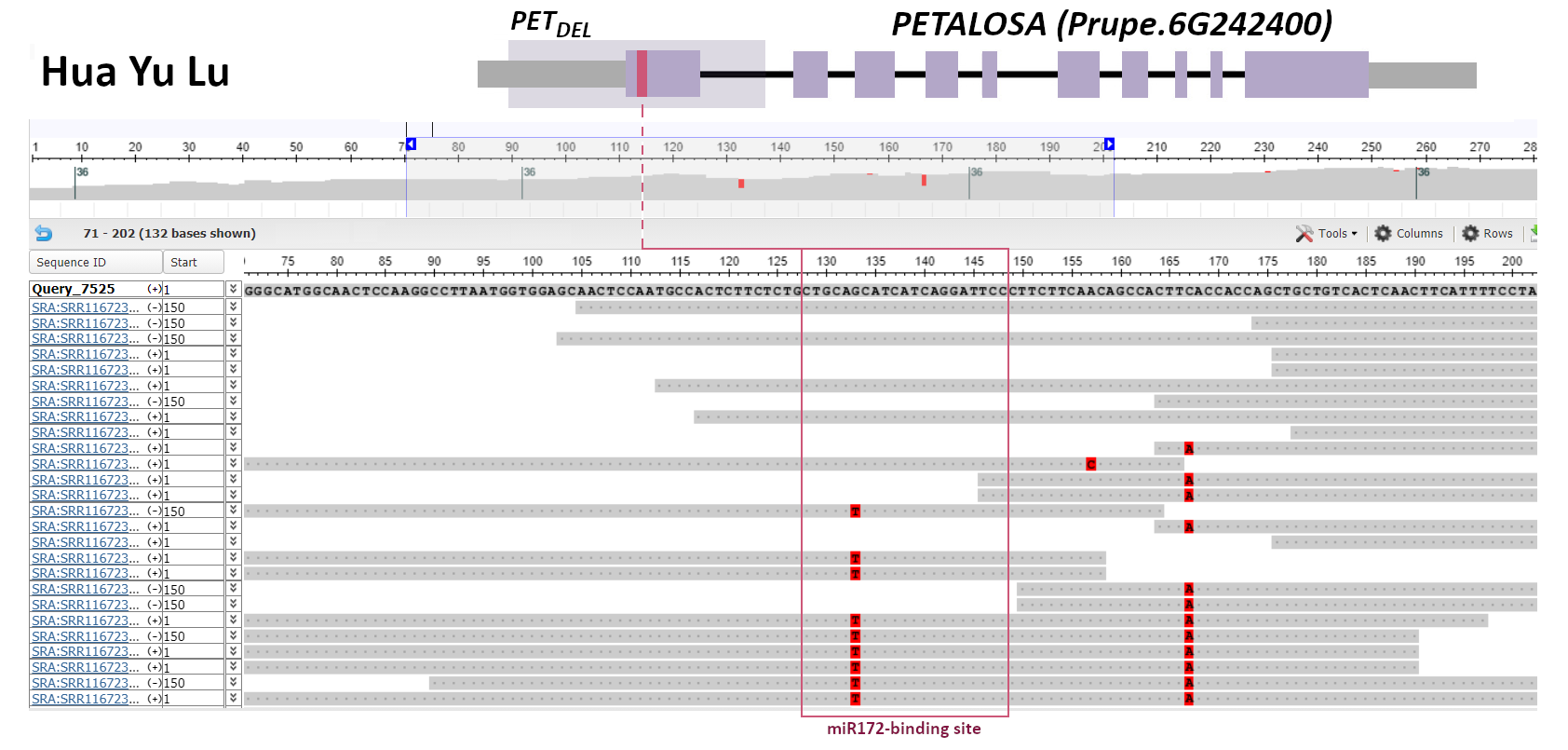

Supplement: Supplementary file 4 — Additional file 4: Supplementary Figure S4. Single nucleotide polymorphism detected within the miR172 seed region of PETALOSA gene (Prupe.6G242400) at the Di2 locus in the double-flower ‘Hua Yu Lu’ accessions. The PET deletion variant near the C terminus (Pp06:24,074,355 - 24,075,350) is also indicated. [file 12870_2022_3691_MOESM4_ESM.png]

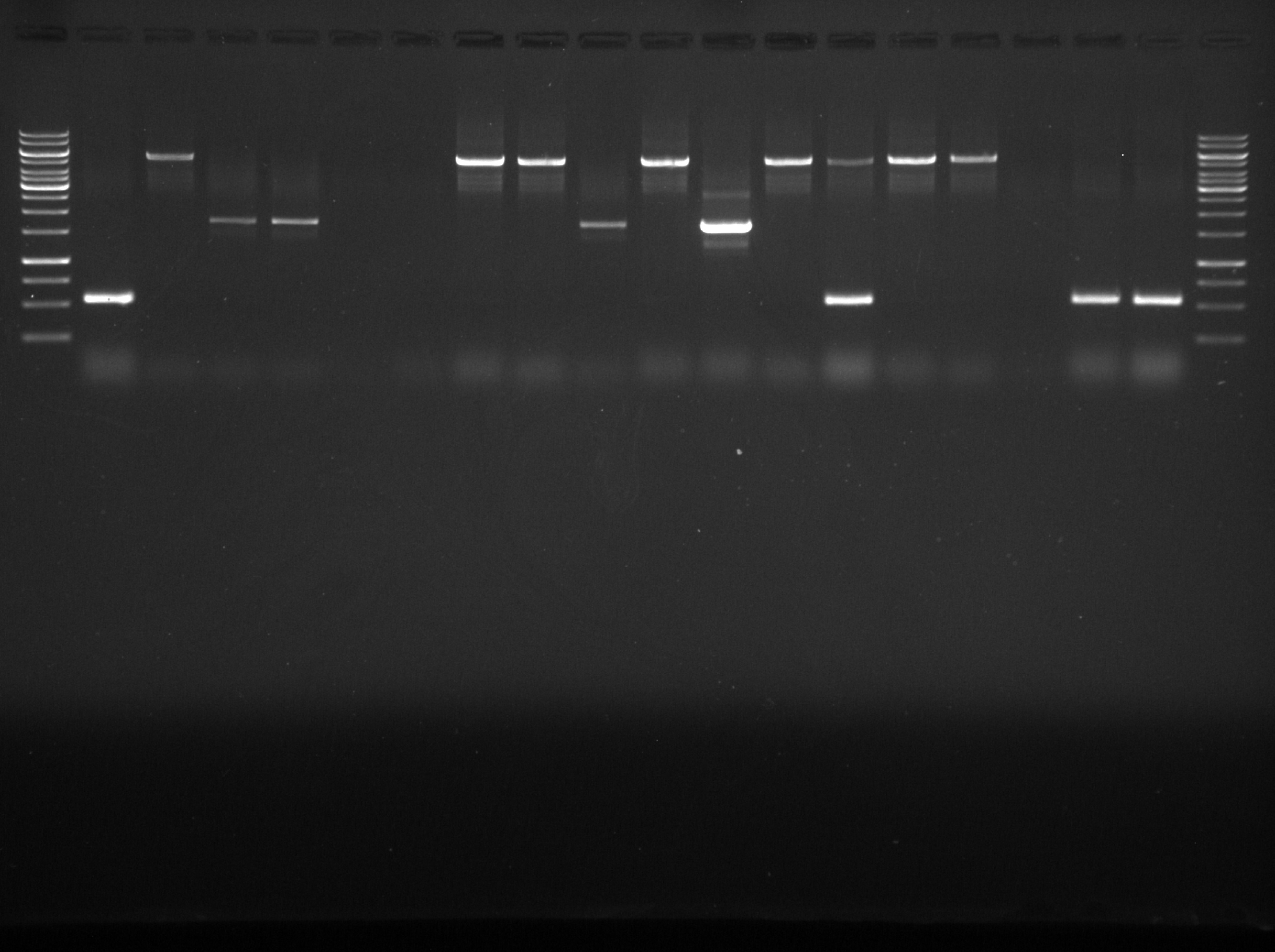

Supplement: Supplementary file 5 — Additional file 5: Supplementary Figure S5. Picture of the agarose gel electrophoresis of PCR products used in Fig. 2. [file 12870_2022_3691_MOESM5_ESM.jpg]

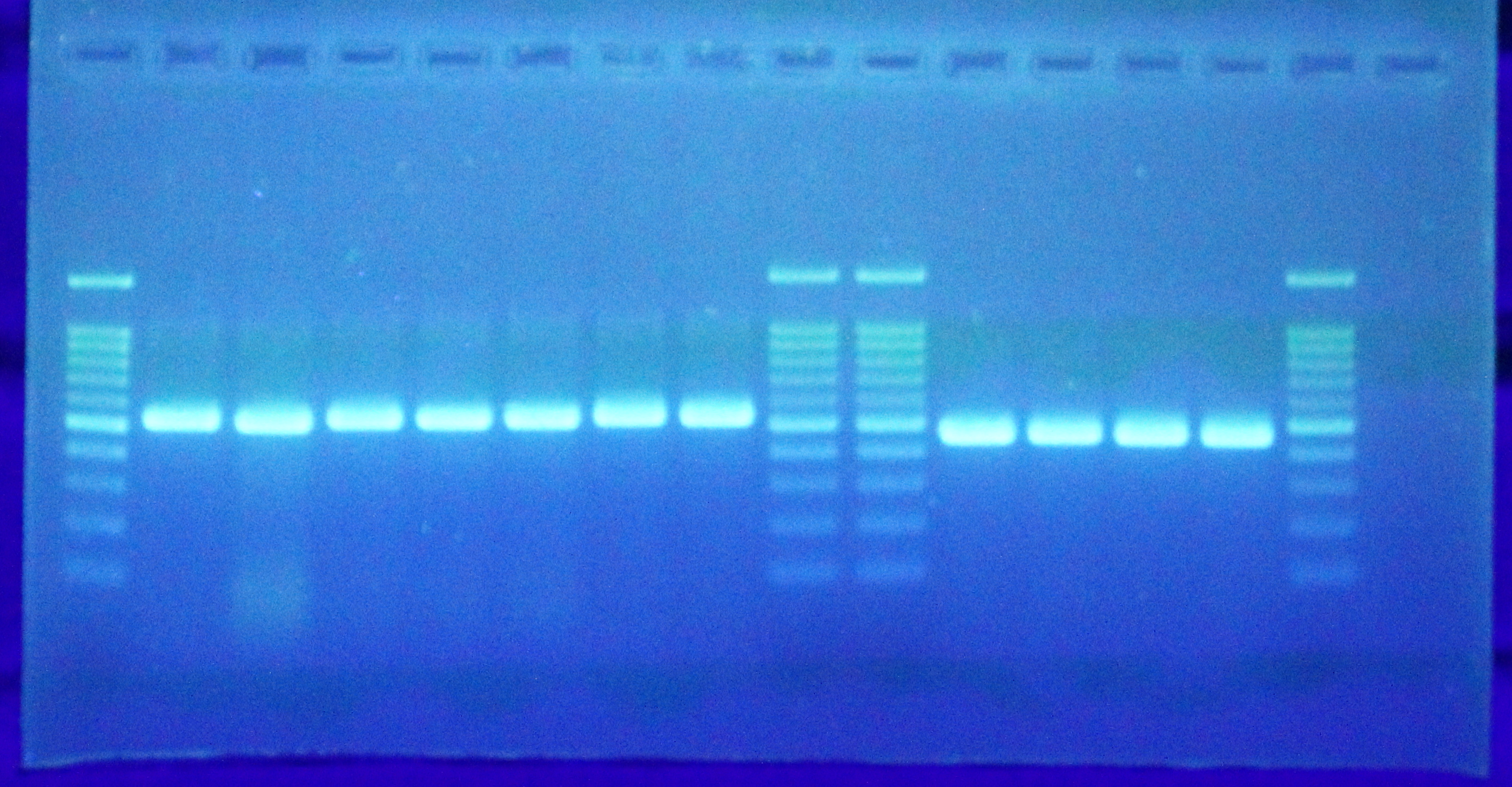

Supplement: Supplementary file 6 — Additional file 6: Supplementary Figure S6. Picture of the agarose gel electrophoresis of PCR products used in Fig. S2. [file 12870_2022_3691_MOESM6_ESM.jpg]
