# Supplementary material for: Less is more: natural variation disrupting a miR172 gene at the di locus underlies the recessive double-flower trait in peach (P. persica L. Batsch)
Source: BMC Plant Biol. 2022 Jul 4;22:318. doi: 10.1186/s12870-022-03691-w (PMC9252053; doi:10.1186/s12870-022-03691-w)
Supplement: Supplementary file 7 — Additional file 7: Supplementary File S1. DNA sequence of the diΔ, di1 and di2 variants. Prupe.2G237700 is highlighted in green with the boxed pre-miR172 sequence. The deletion is indicated in diΔ with strikethrough text while the insertions in di1 and di2 are highlighted in orange and yellow, respectively. Primers used for genotyping are marked as bold, underlined sequences. Primers used for RT-qPCR analysis are shown on a portion of the pre-miR172d sequence. [file 12870_2022_3691_MOESM7_ESM.docx]

**>*di^Δ^***

GCAAAGGGGCATTTTAAAGCACTTACAATGGTTTTCCAGTGAAGAGAGATGCAGTTGCACCTTCTTGTGTCTTAATAGGCCCGCCACTAAGAGTAAGGATACACA

*diDEL_A_For*

AAATCCCTAAACTCTATCGATATTGTATCAATAATTATCGATGACATATTGATAGTTAGATCTTTATTAAATACCAGATATCTTGTA**CAACATTGGATTACACACACTTC**AAAGCAAAAACGAAGAAGAAAAAGTGGGGTGGAAATTTTGAGATAGTGTGTTGGGAAGATAGGAGAGAAAAAGGGGAGTTGAAATGAGGCTATACGAGGACATTGTGGTTTCTAAATGGGGTAAGGCTATACAAGTGCAAATTGCAATCCAGCGTAGCCTACGCCTATATCAAACAGTCCTCAGCGAGATTTTGGGGCTTCTTTTTTCTGACAACTCATTTCAGATTTCAGAAGCGCCCCAAGACCAGAGGGCAAAGTAGAATT~~GTAGCAGCTAGGTTTAGGGTTTCTTTTACTGCTTCCTTTCCTTTTA~~

*diDELwt_D_Rev*

~~ACAAATAAAAAGATTATAATTAA~~**~~GTCTTGGAATCAAGTTTCTGACA~~**~~TAAAATATGAGAGTGCATTATGGGAGAAATTTTATTTAACTTAAGAAGATTAATGTTAAACAAGTAATTAACTATGCCTCTTCTTGGTTGGCCCATCCTTTTAAGAATCTGGTATTATTAAGAAATGAAATTAAGGGGAGAAAAAGGAATAAAGAAGATGGACTACTTGCTTGGAGTTGGAGTTGGAGGGGTGGAGGTAGTGCTTGTGGCTTTTAATTTCGTCCGAAAAGGAGACAATGTTTGTTTGTAGGGCACTAGAAAAGCACGTAGCCCCCACTGGCCACTGGTTTCAAAGTAATATATCTCAGCAAATCAGTAAACCAAAGTTCCCTCTCTCTCTCTCTCTCTCTCTCTCTCTCTCTCTCTCTTCCCCCATCCCTATAATTTATATGAGCTGTTTTAGCTGTGAGAGAGATTCATATATCATTGAGCTGCATGTGACGACAATCCAAGTCACCATGATCTCTAAAGTTTTCATATAAAAAAAATTAAAATTAAAATTAAATTCTTTTTAAGGTTCAAAAAGCAACAAATAATTTGATTTGATAGACATTAACTGGTGATATAAGTCACTGATGAGGAGGATATGGGTGGCTGCGGCGCCGATTTTTTTAAATTTTTTTAATTTTATGAAATAAGGGGGAAGAACCCCATTTTTTCAGAGATTTTAATGATCAAATAGGTTGAACATATAATTGTGCATGATTAAGTTAAGACCTTTGATGGTTCAGATAAAAAAGAGCCACTATATTTATGTGTGTACAAATCATCAGCCTCTTCACGAAATCAGGGTTTATTCCCAATTTAGAGGAACTTAATGAGGATGGTACAAAGTTTGATACTCTAAATGCATCGGTAATTGAGACATTGTAATTTTTAAAAACGCAATATAAGATTTGATCATATTTTAAATAGGCAGTATAATATAATTAAACTTAAAGAATATTAGTGCTTACTGAATTATTTATTATATGATTCACATTAGTTATTTGAGTTATGAATTTTCCCTACAAGAGGAAACAAAGTAGTGGTGATTAAATTTCAGTAATCTACATTGTAGTAGATTATTGTAAAATATAATAAAACGTAAAATAAAGTAATATTATATTATATATAGTGTAATACACAGTCATATATATATATATATATTTTTTTTAAGAAAGTAATTAATTAATAAAATTGTGTTGATCTAAGAGCTAATTAGCAGCAGAACCTCAAAAACCCGTACGAGGTGGTCTCAAGAAGTTTTGCGTTGCTTGCTTTGATCCACATCTCCGTGTTCTCCGCCCCTCCTCTCTCTCTCTCTCTCTCTCTCTCTCTCTCTCCTCTTTGCTCTCCGTCCGTACAGCTGCAGCCATATCAATATCACATCAACTCTTTTTCATATATTGTTATCAGATTTCCGGCCCCTCTCACTCATTAAAAAACTCATGTAATAGTTTTTTTTTCTTTACTTTTGGGACATAAATTTGATTCTCGTTTTCTAATTTTGTTTTTTCTCTCTTTTCTAATTAGACATGGAAATTTGTGACTCTTAATGCAAGTGATATTTAAAAAAATTCTCAATTTCTTGGGAGTCCTACAAGATTCATTTGTTCTTTTTTCTCTAACAGATGGTTAGAACTTCATCTGGATTTGAATCATACTGAAAGGTCCACTAGAGATTAGAAATTGTTGAAGAAAGAACAAGATGTTTCTTTTGTCCCTTCCAGGCGATCGGAAAATAAAGAAATAGTTAATATATTCAAGATAATTATATATTTACAAAATACCGTCCCAATTCATCCTATTTCATTAGATCCGGGATGTGATATGGTTCCGAAGTAAGAACTAGATATGGACAGTTCCAATAAGATTTCATTCTTGAACAAAAATTCATTTTGATTTATTTCATCTATTCCATGACCGGAATAGGGGGGATACACATTACACCACGATTTTGAATCAAAAGAGAGATTTCAAGAAATGACATATAGAGATTTCAAGAAATGGCATATCTATTCACTCTATCAATAACTGAGCCGGATCTAGTGTATCATAAGGGATTTACCTTTTCTATTGGATTGGATCAAAAACAATTCTTGAATGAGGTATTCAACAAGGATGGATCGAAAAAGAAATCTTTATTGATTCTACCTCCTATTTTTTGTGGAGAGAATGAATCTTTTTATCGAAGGATCAGAAAAAAAAAAATAATTCCAAACCTCCTGCGGGAATCATAGTTGAAAGATTATCATTCCTATACTTCCGGTTCAATTTTCCCAAACTAGAACGATGTTTGTTTTAACTTTCAGCAAAAGACAACATTTTTATATGCATACTTGGGTTTGTTTTGTATGTTTTGTGTAACTTAACTAATGTGTGTGTATATAAAAAATCAAGAGTATATGTGTAAAACAACATTAAAACTAGGCTTGACGAAAGAAAAGTTAAGAAGACACTCAAGAATTAATATATGAATCTCCCCTTCTTCTTTAGAGCATCCAGGTTCGATTTACGTATAAAATAGTTTCGAGGCAACTCAATATAATTCTACTCCCTCTCCCTCCTTAGTGAATGACCAAAAATAAAAAAAATAAAAAAATCATTAAATAATAAGAAAATGTTCTTGAAGTGAAATAAAATTTGGGGCTCGATTAAAGCTTATCTTAAAGCCCGAAATTATGTTTTAGCTTCCAAAGTATTATTTTTCTTGGTTGAAAAAGATATTTTAATCTTAAGGAATCGCCCCTTTCACACGTATTGCGCATGTCCTGTATATATGCAAGTCATAGGATAGAATAGATCCAAAAGTTGTAATATCAAGATTCGTGTAATCTTATCTTAATTAATCCACTAATGAGTATGATTACAACTTTCTCAAAGTTTGATGAATCGACACATAATGAGCAAAGGGAGCTTGGAGGAGGTGGAGGTGGAGGACAATAGCATTTAATTATTGTACGTATATCAACCGAGAGGAAAACCTATTGATTCGAAAAAGAAGTTAATAAATAAATAAAATTTTAAATTAAAGTCGAGAGGTGTATTCAATCACGATTTTGTTGATATTGATCGTATTGTAAATTATTCTTGTTGGGTATATTTCTAACAACGACATGTAATTAACACAAAGTAAAACTTGATTGGGATTGGCTTCAAATTCCAGATGCGTATCGCATAAATTTTGGCAGGGAAATGGGAGATGGATCTTTCAGAAAATGACCAGCATGGTAAGACGCACTGCACATGTCCAAATTCGTTTTTGGACCACTGCAGCTGTGCCCCTCTCCCCATTGAAAGTGGGAAAATTGCTTATTCAGACGACTTTTTGAGTCGTTCCAATGTCCGATTCAAGATCTTACAGCTCACAATAATAATTAACATGTATGAATTGTGGTTCATGTCAAGCCAGGGAGCAAGAGAACAAAAAAAATAACCTTACATAAGAGGAAAGGGGTGGGGATCAGAAGCCTCTAATTACTATAATTACTATGGGGCGTATTGCAAGATAAGTTGATGAGATATAAATGCTCCACGTCTACTGGAGTAAAGAGATTGAAGGAAACATAACTCATATCCACCAGTAAAATTGAGTGTTTCAAAATGCAGATGTGGGTTACGCCAGTTGATTAGGACAATGAATTTGTATTTTTAAACTAAAGTTTGAATCTAAATCTAGTAGATTAGATTAATTTATTATCATTTGTATTAAAAAAAACCATAAATGAATCAAAATCATCAAAGGGCATTGCCTCTCATCCACCAAAGAAAAAAGTGGAATGTAGTGAATGCATGACAAAGTACTTAACCCTTTAGAATTACAAGGAGTTTTTCATTTTTGGAAATTATGGGTGCACCAAGTTAACATAAGAGCTAGAGCTAGCTAGACTAGCTGGCTTATAAAACTAATTAAGATGGATTAGACATGTATATATATAGGGCAGTCTCTTAAAGAATCCAAAAGTGGGAAATGAGGGATCAACACAAAGCAACCTTAAACCCTTAAAACCACCCAACACCCCCCCACACCCCCCCCACACACATTAGTCTAGATAAGGTCATTCATGAAGGTCAGGGGTCTGGGTGAAGGGGGCTTGTCAATTGCCTCCCCACCCCACTCAAAGAAATATGCTTGTAGAAGACGCAACATATTTAACATCCAAACCTAAACGCCACCCCAAAAATATCAACAATATGTATTTGAACGATGATGATCAAGTACTACTGGTTAAGCTTTCATTTTCCAACAACATATACATATATACATATATAGGTTTAATTAAATAATTGCAGAGAGAGGTAGCTTTTAATTTTATTCTGGAGACATCTGTGGAACACAGACTGGGTCACAGAAGATTAAAGTTTTTGGTCAATTTGGTACCCAAAGATTACGATTGATAACTGTTACTCCTCATATTGTGCCCAATAGCTTCTCTCTCTCTCCCCCTGACAAATCTCTAATCACCAACCACACACACACACACACACTCTCTCTCTCTCTCTCTCTCTCTCTCTCTCTCTCATTAAATCATTTCTCCTACCCCAGTGAAGCTCTATATATTCATGCCCTCCAACCCTCACCAGTCTCTCAATCTTCTTCAACCTCCTCCTCTACTTCTACTTCCCTGTGAATCCCTTCCTTTCCCCTTTTTCACTTTTTCATAGTCGGTTTAACCACAGAGAAACAACTATATACAAGGCCAGCTTTTCCCTTTCTTTATCCGTTTTACCATGTGGTCCCTGGGTCCTTTATATAATTATTCATAAATATATGTAGACCTACACATGGTGATGGTGGTTTCAACTTTCATTTTGGGCTTTTCTTTTATGTCTTTCGTTTTCAGCCTTAATAACTTGTATAGAGTCCTCCTCCCTCCCTCCCCTTTTCTGTCCTTTACTTTTCTCTCTTGCGTTATATCATGAGATTTAGGGTTAGATCATCAGCTAGCTAGGGTTTCCTTGTCCTTGTTTCGATTCGATCTATTTCGCTTTAATCTCTCTTTTTGCATGGGCGTGATGATATCATGAGGATGGAGATGGCAAAGACAAAGAGATCCACACAAAAGAAAGACAAAAACCAGTCATTGTTTGCGGATGGAGCATCATCAAGATTCACAATTTCTTGGGGCTAGCTGCTTTGCTATTGGCCCTTTGATGATATGGGAATCTTGATGATGCTGCAGCGGCAATAAATGGCTATATATATACACTCACTCTTCACCCCAATAGCTCATGAATCATGAACATCACACTAAACCATTCAG~~*~~GTAAAACATTTAGATACTGGTCTTTCTTCTTTCTTTTTCTTTTTTGTAATTTTTGAGATGGATGGTGATGGTGAATTTATTAATTATATGAGAAGCACATTGCTGTTTGTTTTGACAATGATGCTTCTTTTCCTATTGGCCTGTTGATTTGTTTTATCTAATGCACCATGTATTTACAATGTTTAATTAAGGATATATATTGGTTTGGGGTTTGTGTTTGTTTGTCTTTGCTTCTTTCTCCCACTTGCACCATTTCGAGTTCTGCAATTGTTCTGTCGTTATCGAGAAAATAAGGGGCGAGTGGGGACTTGGGTTGGGAGGGCAATATCCATTTCATGTGGTAAATACCATTCTGTACTTGTTTAATATATAACTCGAGGTTGTAATTACTTTTAGCTTCGAGCTTTTTCCAG~~*~~GGAGTGAAGATTTCTCAGAGTTTTGCAGTTCTGGAACTGCAACAGCCCTGATCTCAGGAATGTCTGTACTTTGCTCGAAAATGTTACGCCATATACAAGAAATCATGGGCCTTTTGATTTCGTTTTTTGAAAACTTTCCAAGCACTCTTTAGATATTTTCTCTTTTATTTATTTATTATTATTTGGTTTTTTCTTTTGTTTTTGCTTTTCTAACTCTAGATCCATCACCTTCATGATGGTCTCCATTTTGGAATTACTCTATGCCAGCAGGTTTCAGATTTTGGTGACATGATTTCTTTGGAATATGAGATCAAAGTCTCTCATACAATCATCGCTTGCAGCTGTGTCCAGTTTTGGTTTGTCTGCTTCTCTTTGCTCTCTCTACCACTTTTGTCATGCCGGACAAGACGAGCCCTCATCTACTTATGCCATTCTTTCCCATTTAGAGTTTTTCCAACATTCTTCTTATTTTATCTGTAATCTTTGTGCTCTATCATGTATTAGAAAATATTTTTGATTTCAAGTTTTATTTATCCAACGGGTTGATCTTCGGCGCTGTTGTCATCGCCGTCTAGGGTTTGGAGAAGCCTACTCCATGGGGTAAATGTTATGTGTAATGGCTACTCAAACTCAAATGTATTAGCTCTGTATTTACTCACCTGCTTTGATAAAAACTTTGTAATTGAATCTTTCTTGTTTTGTTCTCTTATTGTTGCGGTCATTTTACTGCTTCTACCACTGCCAGATGTAAAAATTGTGTGAAGTTGTCATTATTTATATCAAAGCTCTATTTTTTGTTGGTCAGTGTGAGCTTCGTCTTATTTGATGTGCTATATAATTACACAGATATGTAGTAATATTAACACGTGGGAGAATATTATGAAGGGAAGCATTGTTTTATAGCATTAGTTTTTTTTTGCACATTATGTGGTAGTCGGGTTCATTGGACATGAATGCATCTAGTATGTTTGAAAGTCAGATTTGATACACAAACAAGCGATCTTGGGAAAGGGAAGAATCGAAGATAAGACTCCGGGTGCATAGACGCCTTGAAAATATTGGAAGTCAGATTTCAGTCCAATTTGTTGTTTGGTATTTATAGCAGTAATTCTTTGTCATCTTCTTTCTTTGTAGGAGTTCGTAGAAAATATTGTAAAGAAAAGTGCTATCTCGTTTTTCTTCCCCAAAACCAAGTGATGCGTTTTCAATCACAACATGATGATAGATACCTAATCAAGGAATGTACCTGACTTGCTGATATTAATTCTTAGTCAAAGAAATTGATATGAGTCTGCACAAGTTGGTGAGAAATGACCACAGTTGGGTTCCATGTTTTCGTCTTAAATGAGTCACAAATAGGGACTAGATTTTGGACTTCCTTTGAAAGATACAAGTTGTGATCTTGTTGGGTTTGTCTGTATCTGGGCCAGCACTATGAAGCCTACAAGAGAGATCATCAGAGATTTGGGGTGGGCTTTCATCTTTGATGTTAAAGGGCCTAATTACATTGGGTCTTGTTGGTTTCTTTATGTAATTGTAGGGAGGAATGAAACATCTTTACATTACCTATATGCATGATCTTAGCAAAAGATGATATGAGGAAAGGGAACAAGATCTCTTCCAATCATATGATCATAATCAACCATAAGATCACAGAATATTAGGCAAGAATAAGCAACAAAATCAAATGATTATAATCGACTTAATCATCTTATACGATTCCTTGTCGCTCTAATTCGTTTTGATTAATTCGATTAATTTGTTTAGGTATGGATAGCAATACGAAAAACAAGTATTCATTTTTTGAACAAAAATATGGGGAATGGTGTCTCATCTCATGTCCCCTCATTTTTTGAACAAAAATATGAGTTTTGGTTGGTTTTTAGTTGTTGTCTTAGTTCTTTGGTTTGTTTTTTTAATGTTATGCGAGCCTCTGGCCTTTGGAGAGGTTTGGAACATGTAGCTCACATGGAGGCATGTGATGTAAAATAGTCATCTCTTCCCACTTTGATGAAATGAAACAGACAATCAATATATATGTATAGCAAACTTGACCTCATTAAGGAATAAAATCTTATTCTGCTATAGCACAATTAGCAGCGTTTCATATATATATAGAAAACCATTCTGATAAATTTATATACATGAGAGGCAGCCAGATAAACCAGAGAATATATTTTCTCAGTCAAACAGACACAATCAGGTGATCCCAGTTGGAACCTTGTGCAATGCTAAAGCAGAGAGGTTGGTAAATTATAGACCTCTCTGCTTCTGCTTTTCACATGCCCTGATTGTCTTAGAAAGTATCTTTCAACCCAGTTGATAAAAACAAACCATGGATTGTGTTGAACAGATAGTTAATCCAGGATAATATCATTATTCTTATTCTCCATTGCTGTAATAAATTTTAATTTTGCAATATGCATATCTCAAGGTTCCAAAACCAAGATTGAAGATTCTGTTTGTTATTTTGACGTCTGCATTGTGCAAATCTTATGGTATCTTTCATTTAGCTGTTTGATTTTTTCAAGTTGATGGCATCATCCACTAAGCATAAAGAAGTGAAAAACTTCTTTAACCTCATTTCAATCATGAGCACTAGTCACAAGCAAAGAAGAAAGCTAGCATAAAGATGCGTCATTCCAATTAGCATCAGAAAGATTTGAAGGGTGTATGTTCCCTCTCATGCAATTAGTAACATGGCATATCTTAAGGGAGCTGCTCCTCATTTTAATTTGGAGCTGCTTCATGATCGGTTTTGGTGCAACAGAAAAAAAGGAGGGACCTGTGGATTGTTCATAACGTATCGAACTTTTGGTTCGTAAAAAGAAAGTATTATATGCAGGGTTTGTTATAATTAAACATAAAGCATATATTGCTGCAAAAGAAGAGTGAGTTGAAGAAATCTGTGTCTGGGATTAGGTGCATTCGAGTAAAAAGTACCAGTTATCATAACATAGAAATATAGAGTGCAAGCCAATCAATCTAAAGTTCAAAAATCAACACTTGGGAGTGTAGCAAGGAAACTTTGCTGCTTCACTGAAGTTTTGACTGATAAACAACTCAATGCCTGGCTTAACACAGACAACGTGTTCTTGGTTCATCTAGTACAATCATCCCCAAAGTGCATATGGTTCTCTTGTTTTGCTCTCTTTAATTTGTTCACAGAACCAAAGTTGCCTGCTTATGCATTGCAGTTCTTATATCGCTTAGAAGTAGAGCAGTTGCTTTGTCCCGGCTCCTTCAGAAAAATAATGAATCACATAACCACATTTCCATGCTTGTCACAACAGTGGAGACAAAATATTGATGAGCTTAATCAGAAAAAATAAAAAATTCAATATTGATCTAGAAGAACATGCAGCAAATCTTAGTGAGTATCTGTAGTAAGGGTATTTTTGATTCCGAGAAGATACAATGAACATGGAGATACAAATCGTCATCATAATTATCATCAGGTCACAGAATCAATCAAGAAGTTAGTGGAAAAGAAAAAATGGGTGCAATCAAATTAAACAATTTCCTGCTGCTTCATATCATATACATTACTGTTTATATATGCATATACTTGCAGAAAAATAAGAAGGTAATTTAGTTTAAGAAAAGATAAGTTAGATACATTTTAGTGACCCTCTCACTT~~

*diDELwt_C_For*

~~TCTCTGTCT~~**~~CGCTGATATTTGAAGGGTTTCAC~~**~~TGTGTTTTGCAGGCTTGCTGGCAGATACACACACATTACACATAATAAATATGAAGAAGGTTTGATATTATTAAGCAAGCCATGATTAAGTG~~TATTATCTGCATGGCTAAACACAGCACAAGCGATAATGTTGGAAGCTTATTGACCCAAGACAGATTTTGCAGCATTAATTAAGGGA

*diDEL_B_Rev*

GAATTATTAGTTTTATTTGCCTTATGATTAAATCATTCTTTGTTTTTGACAATTTAAAGCTTTCTAGAGGC**GCCACTACAGTTTATACTGGACA**CAACAGCAGATTTTCTCATATATAATTATTCAAATAAATATTGACTAAATAAAGATTTGCTTGAACAATCAAATGCAAAACAGAATAAGTAATATAATTAACCAAAGTGTGAATACTACAGAAATGTCTAAACCATCAGAATTCAGAAGCATTCTTATTTGTTATATAAACGTTAAATTTGATGTTGAAAAGAAGACATGGAGACAACTGAGAATCATATCAATTAGAAAACTAAAGCTAAGCTTAGTCTAGGGGCCAAGAGGCAACAGATTATTAAGATTCCATATATATATATATATATATATAGAGAGAGAGAGAGAGAGAGAGAGAGAGAGGAATATATAAGCTCTGCTATTTTGCCTCCATCAAAATTGAAAAGCTATATTGTTAGTGGTCCCAAACCTTGAATTTGAAAAGTAGAAACAGAAAGGAGAAGAACCGAGTAGGACAAACTGAATTAATCAATTGCAAAACTTGATTAATTAAGTCTGATTAAGACTGAATTGACTAACTACCCATGATCCTAGATGCCTGCTGATTTAGGTTCTAGACTCTTTAATTGGGTTCTTATTCATG

**>*di^1^***

acttcCCTGTGAATCCCTTCCTTTCCCCTTTTTCACTTTTTCATAGTCGGTTTAACCACAGAGAAACAACTATATACAAGGCCAGCTTTTCCCTTTCTTTATCC**G**

*2G237700_F1*

**TTTTACCATGTGGTCCCTGGG**TCCTTTATATAATTATTCATAAATATATGTAGACCTACACATGGTGATGGTGGTTTCAACTTTCATTTTGGGCTTTTCTTTTAGG

*2G237700_di1_Rev*

GTAATGCTAGGGAGACCATCTAGTTGTACCATATTTTTGTA**CCATCTGATGTGGCTGATGAGT**TGGCTATAATTTTTTAATTTTTAATTTGTACCATATTAAATGAGCTTTTAATTTTTAATTTTTAATTTTATTATCTCTTCACTTTACGTAAACCTAAGCGGTGTAGGGAAGTTTTGTTTCACGTATATTCAATGGTTCTTCCTTACCTCTTCCCCTTCCTCATCCTTATCTTCTCTCTAATTTGCCAACGACGATAAATCATACTGCAGTTTTTCCTGACGCCTGTGACGACGGTAAGTTCATCTCTATTTCACTCTTAGGTTTTCTCTTGTTGGGTGATGATTGAAATTTTTGGATTGATTCTTTGAATCATTGAGATATTTGTATTACTCTTACGATCGGTATTGAATACTTTCATACTAATTGTATATCATGTTCAATATTTGTTTTGGGTCTCATAACTAGCCGTTTTTGGTTCTTTCTACGATGGCTTGACATGCCTCCCTCCATACTTGAAGTCTTTGATTTTTTTTTTTTTTTTCTTGTTTTGAGTAAATCTATGTTTACTGGGAGTTTTGTTTTCTCCTATCCGTATTCCCTTTTTGTGTCTTGTGTGTTGTGTTTGTTTTCTCCTATGCATATGGCACTCATGCAATTTTGCAAGTTCAAATAAAATACCATGGCTATAATATTACAATGGTTGCTGTGTTGAAACCTCTTTTTCCTTGCTTGTATGGATATGGGTCTTTGTAATTTATGTTGGTTGCTGTTTTGAAATCTCTTTTTCCATGCCTTCCTTTTTCTTCTTCTCACTTTACAGAGATTCTAAGCAGCTGTGATATGGCATCATTCCAAGTATTCTTGAATGTCCATTGGTCCAAAAACGACTCACTTTTGGGGACCCGTTGCAAACTGATTTGTTTCCGTTGTATGCTTCACTTCTATGATATATAGAATGTATTTGATTTCAAAATGAGGTTTCTTGCTTTTTGACTAATTCTGATCATAATGGGCTGATTTAATCAGGATTGACGGATATGAACAAACCTCTAAAAATGATTTCTCGGCAACAAGACAACGAGTCCAATCTCAAACTAAAAGGGTGGCACCTAATTCAATCAGAGGTTGTAGCAACCTTAATCCAATCTCAAACTACAACCATTCAATCTCAAGTGCAATATGGTCGTTTCAATTACAACACTAACAGCAAAATGTCTACATTAACAAAAAAAAAGGAATTTTTAAGTTCAAAAGTGTCAACAACTTCTGCAATTGCAATTCTTTTGCTTCCAATGATCGATTTGTTGGCCACCACTTCCTCCAGAACTAAAGCTAGGCATGTATATATTGTAGTCTTCATTCCAATATTGATATTGATGATCCTAAATATGAAAAAAAAAAAAAAAAGGAAGAGGAAACAATATATAAATTCAACATTTTAATATAAAATAAATAAAAAGTACGGAAAGTTAGAAATTAAACTTCTTTTCTATTACCAGAAAATGATTCTCATGAAGGATTGGATGAGCCATATGTCCTATCTCATACTCTCTATACAACTGATTTAATTGACCAGTTTGAGTAAAACCCATAAATGTGGTCATATTTTTCCGCAATGTTTCCATAATATTGGTCGTTACAATTAGCGAATCATCGGTACTAGAGCAAATGACTAATAATGAATTTTTAACAAACAAAAACAATCAATCAAAACAAAACATTCATGTCATACCCTGATAGCAATGCTCTTTGTGTTCCAACTACACCATCACCTTTAGAAGTAGATGAACCAACAATATGGCCAATATCATCAAATTACATTTGGCTGCAAATCCTGCATAAAAACGCATAAAAACTCACAATAAAATACTTATTGATTGAATAATACATTAGAAAATAATAATAAAAAATGTATCCATTTTAATTGCATACATTCAGTTCTTTCTCTCCATTAGTGTTGTTTCCACAAGATTTGATCTTCTTGTTACTCTCTTGTTTCTTCTTTTTACTATCTTGTTTCTTCCTAAACTGCTTGTTCCAACGAGATTTCCTCCTTTTAAATGGTGGACGGCCTTTCTTTCTAGCTGCTAAGGGAGTAAGAATACATTGACTTGCATTCGGAATTGAACTAACATCATTCTCAATGTTCCCGCCAATAGGTGACCGAGGATTTGGTTGATCACTACCACAAACAACATTATGTTTGGAGAGTTCCTCCTTTACATTATCCATCCAAGTCATCACAATCATGCACTTATTGTCAGAATCATTTGCCAATTCCTTAATCTCATCAAAAGCCTTTTGCATCTTATGACATCGTTGTGCTTCAGTGCGAGACTATAGCTTTCATAATTGATTTCAATCCTTGTGTGACATCTTTTCACATCCTTCCTCCATCTTCGCATTATATATTTTTCTGGAATCAAGTCAATGTTGTGGCGAAAGAAAAACATATATTGTATGACGGCATAATATGCCCTAAACTCAAACATGCGACAATTGCACTGACTTCATTGTCATCTTCATTAAGCCAAACCTTGAAATAAAGCCTCTTTTTTCCCTTCCATCATGAAATCCTCAGATATTTGGTACTCAAAATTTGCACCATTCACTTTATGTGAAACATAGTCGCAATACATTTTGCCTATTAATTCTTCATGAAATTCTTTATATTTGGAAATAGTGTAAACCCTTGAGCCTCGTTTCTCCATGTTATAATGAGTTCCACAATCAAAACCAATGGATGAAGACTTAAAATCTTCTTGCTTCTCCTTTTCCACCTTAACTTTCAATGCATTTTCATATTGCTCCACAAACTGTTTTAAAGTAGTCTTAGAATTTACATGGTCATCAAAAATGCATTCATACTTTCACTTCGTTGTGTGGTAGACATTCCCGCCCAAAAACTATCTTTCACAAAATATGGTACCCAACGTTGTCTCTCATTATATAGTCCAGCCAACCATTCATTGTCTTGTAACTCATATTTCTCAATTATTTCATCCCAACCTTCTTCAAACTCAACCTTCTCCAATGAGCCATACACAATACTTTGCAAGGCATACACTGATAGCTTCATATTGCTTGTAACGGTTCAACTTACTAGGAAACTTGTTCATTATGTGCCACAAGCACCAACGATGTCTAGTATTAGGAAAAATAATCTCAATGGCATTTTTCATGGCTTTATCTTGATCAGTAATTATTCCCCTAGGAGCGTGCTCATGCATACATGTGAGCCAAGACTTAAACAACCAAATAAAAGCATCAGTATCTTCACTTGAAATCAGCCCACATCCAAGTAAAATTGATTGCCCATGATGATTTACCCCCACAAATGGAGCAAATGGCATGTCATACTTATTCGTCAAGTAGGTTGTATCAAATGTAACAACATCTCCAAATCCTTATATGCCGCCTACTCTTGCATCTGCCCAAAATACATTTCTTAACCGACCATCTTGATCTAAATCAATTGCATAGAAGAAATTTGAATTTTGAGCTTGCATATTCAGAAAATAAGTTTGGATTGCAGTAGCATCGCCTTCCCCAAGCCGTAATCGTCTCACATTTTCAATATAATTTCTACAATCCTTTTCCAAAAAGGAGATATTCTCATGGCCCCCACCAACCACTAATGAATTATAGCTCTTGTTTAACCTTATTCCAGCTCTATCATTTAACTCAATCCTCCTTTTCATATATGAACTTATTGTCCGATGACATCGAAAATAACGAGCCTTGGTTGGACTCATATCATGGTTATGATCAAGGTTGATAGAGTTGATCTGCCACCTTCCATCTAAGATTATACCTGCTCTTACTCTAGCTTTGCAACCAATTTTTATACTTGGATGTGGCTTTAGGACATTACTTGATTTACTCTTATATTTGCCTTCTCGACCACATGAAATAGTCACATATTTCAATTCTCCTTCATCTCCCTTTGTGATGTTCTCTTCCTCACGGGAAACCCTAATTGCTTCCCATATTCTCTATAATAGACAACTATATCATCAATGTTGTTAAAAGTCATCCCAACTCGAGGCTCTTCCACATTCTCTATTCTCCAATTCAGGCTCCTCACTTACCGCTTCACCATCATTCTTTTCCATTTGAACCCCATCTTCTTCTTGATTAATATATGAACTCGTAAAGCTACTCGAAGACATGATTACAAAATAAAATAGGATCTGCATAGTAATGGAAGGCATGGAAAAAGAGATTTCAAAACAGCAACCAACATAAATTACAAAGACCCATATCCATACAAGCAAGGAAAAAGAGGTTTCAACACGGCAACCATTGTAATATTATAGCCATGGTATTTTATTTGAACTTGCAAAATTGCATGAGTGCCATATGCATAGGAGAAAACAAACACAACACACAAGACACAAAAAGGGAATACGGATAGGAGAAAACAAAACTCCCAGTAACATAAGATTACTCAAAAACAAGAAAAAAAAAATCAAAGACTTCAAGTATGGGGGAGGCATGTCAAGCCATCGTAGAAAAGAACCAAAAACGGCCAGTTATGAGACCCAAAACAATATTGAACATGATATACAATTAGTATGAAAGTATTCAATACCGATCGTAAGAGTAATACAAATATCTCAATGATTCAAAGAATCAATCCAAAAATTTCAATCATCACCCAACAAGAGAAAACCTAAGAGTGAAATAGAGATGAACTTACCGTCGTCACGGGCGGGAAAAACTGTATGATTTATCGTCGTTGGCAAATTAGAGAGAAGATAAGGATGAGGAAGGGAAGAGGTAAGGAAGAACCAGTTGAATATACGTGAAACAAAACTTCCTACACACCGCTTAGGTTTCTGTAAAGTGAAGAGATAATAAAATTAAAAATTAAAAATTAAAAGCTCATTTAATATGGTACAAATTAAAAATTAAAAAATTATCAACCATTGGTACAAAAATATGGTACAACTAGATGGTCTCCCTAGCATTACCCTTTCTTTTATGTCTTTCGTTTTCAGCCTTAATAACTTGTATAGAGTCCTCCTCCCTCCCTCCCCTTTTCTGTCCTTTACTTTTCTCTCTTGCGTTATATCATGAGATTTAGGGTTAGATCATCAGCTAGCTAGGGTTTCCTTGTCCTTGTTTCGATTCGAT

CTATTTCGCTTTAATCTCTCTTTTTGCATGGGCGTGATGATATCATGAGGATGGAGATGGCAAAGACAAAGAGATCCACACAAAAGAAAGACAAAAACCAGTCATTGTTTGCGGATGGAGCATCATCAAGATTCACAATTTCTTGGGGCTAGCTGCTTTGCTATTGGCCCTTTGATGATATGGGAATCTTGATGATGCTGCAGCGGCAAT

*2G237700_R2*

AAATGGCTATATATATACACTCACTCTTCACCCCAATAGCTCATGAATCATGAACATCACACTAAACCATT**CAGGTAAAACATTTAGATACTGGTC**TTTCTTCTTTCTTTTTCTTTTTTGTAATTTTTGAGATGGATGGTGATGGTGAATTTATTAATTATATGAGAAGCACATTGCTGTTTGTTTTGACAATGATGCTTCTTTTCCTATTGGCCTGTTGATTTGTTTTATCTAATGCACCATGTATTTACAATGTTTAATTAAGGATATATATTGGTTTGGGGTTTGTGTTTGTTTGTCTTTGCTTCTTTCTCCCACTTGCACCATTTCGAGTTCTGCAATTGTTCTGTCGTTATCGAGAAAATAAGGGGCGAGTGGGGACTTGGGTTGGGAGGGCAATATCCATTTCATGTGGTAAATACCATTCTGTACTTGTTTAATATATAACTCGAGGTTGTAATTACTTTTAGCTTCGAGCTTTTTCCAGGGAGTGAAGATTTCTCAGAGTTTTGCAGTTCTGGAACTGCAACAGCCCTGATCTCAGGAATGTCTGTACTTTGCTCGAAAATGTTACGCCATATACAAGAAATCATGGGCCTTTTGATTTCGTTTTTTGAAAACTTTCCAAGCACTCTTTAGATATTTTCTCTTTTATTTATTTATTATTATTTGGTTTTTTCTTTTGTTTTTGCTTTTCTAACTCTAGATCCATCACCTTCATGATGGTCTCCATTTTGGAATTACTCTATGCCAGCAGGTTTCAGATTTTGGTGACATGATTTCTTTGGAATATGAGATCAAAGTCTCTCATACAATCATCGCTTGCAGCTGTGTCCAGTTTTGGTTTGTCTGCTTCTCTTTGCTCTCTCTACCACTTTTGTCATGCCGGACAAGACGAGCCCTCATCTACTTATGCCATTCTTTCCCATTTAGAGTTTTTCCAACATTCTTCTTATTTTATCTGTAATCTTTGTGCTCTATCATGTATTAGAAAATATTTTTGATTTCAAGTTTTATTTATCCAACGGGTTGATCTTCGGCGCTGTTGTCATCGCCGTCTAGGGTTTGGAGAAGCCTACTCCATGGGGTAAATGTTATGTGTAATGGCTACTCAAACTCAAATGTATTAGCTCTGTATTTACTCACCTGCTTTGATAAAAACTTTGTAATTGAATCTTTCTTGTTTTGTTCTCTTATTGTTGCGGTCATTTTACTGCTTCTACCACTGCCAGATGTAAAAATTGTGTGAAGTTGTCATTATTTATATCAAAGCTCTATTTTTTGTTGGTCAGTGTGAGCTTCGTCTTATTTGATGTGCTATATAATT

**>*di^2^***

acttcCCTGTGAATCCCTTCCTTTCCCCTTTTTCACTTTTTCATAGTCGGTTTAACCACAGAGAAACAACTATATACAAGGCCAGCTTTTCCCTTTCTTTATCC**G**

*2G237700_F1*

**TTTTACCATGTGGTCCCTGGG**TCCTTTATATAATTATTCATAAATATATGTAGACCTACACATGGTGATGGTGGTTTCAACTTTCATTTTGGGCTTTTCTTTTATGTCTTTCGTTTTCAGCCTTAATAACTTGTATAGAGTCCTCCTCCCTCCCTCCCCTTTTCTGTCCTTTACATATCTTTTTCCGAAAAAGCAAAGTCATTTTAGGGGTCGTTTGGTACGGCGGACTGTTATGGACCGGACTAAATCCTAGGACTGTCTTGGATTAGCTCGGATTGGATTAAGCTGGATTAAGTACTGACCTACGTTTGGTGTTGCGTTGGACTAAAAAACTGGATTGTGAAAATAGTGAAGACTTATGTTTGGTGTTGTGTTGGATAAAAATAATAATTTTTTAAATTTTTTAATTTTAATTAAGAATTTTAGCGTAAAAATAAATAAAATTCTAATATTTTTGAAAGACTAAAACTGTCACATTTCTTTTTATTTTTCTTCATCCATACTCTTCTCTCCTTTGTCTTTTCTTCTTTATCTTTTCTGATTTCTTTTCTTCTTTATCTTTTCTGATTTCTTTTCTTCTTTGTCTTTTCTTATTTCTTTTCTTCTCTGCCCCTTTTTTCCCCCATTTCTTCCTTCATCTTCACTCCTCTGTATTTTCTTCCCAGGCCCGTTTCTTTTTTTTTTTTTTTTTTTCCTTCCTTCATCCTCTCTTCTTCTTTCCATTCCCATGCCATTTCTGTTTTTCTTTGTTTCATTCTTCCTATTCTTTCCTTTATCTTTTCTTCTTCCTTCCCTCCTTCTGACTCTCTCTTCATGCCACTACAGATTCAACCATCAACTCACCCTCTCTCTCTCTCTCTCTCTCTCTCTCTCTCTCTCTCTCTCTCTCTCTCTCTCTCTCTCTCTCTCTCTCTCTCTCTTTCTAGTTCCACTAATTAATGGTTGTGGTGGTTCGATTGGGTAGTGCTGGTGGTTTAACTGGGTATGTGGTGGCTGGTGATGATGGTTTGATTGACTAATTGGGTGATGGTGATGGTTCGATTGGGTATGTGGTGGCTGGTTATGTGTTTGATGGTGGTGGTCTGATGGTTGTGGCATCGTGGTGATGGTGGTATGTTTGATGTTTCGGGCTCGTGTGTTTGATGGTAGAGGTGGTATTCAGCCGGACTGTGTGTTCAAAATAGCATCCTCGCTATTTTGCGAACCCAGTTTTGGGCTCGCTATATTAAACAAGCGAGTCCAACGTTTTTTTTCCCATTG

*2G237700_di2_For*

GACTATATAATCTCATTTAACTTAATCCCTTCCCTTACCAAACATAGGTTTTA**AGGACTATTTAACCCAGTCCAG**TCCAGTGAGGCTTAGTGAGGCATGCCAAACAGGGCCTTTTTCTCTCTTGCGTTATATCATGAGATTTAGGGTTAGATCATCAGCTAGCTAGGGTTTCCTTGTCCTTGTTTCGATTCGATCTATTTCGCTTTAATC

*2G237700_R1*

TCTCTTTTTG**CATGGGCGTGATGATATCATGA**GGATGGAGATGGcaaagacaaagagatccacacaaaagaaagacaaaaacCAGTCATTGTTTGCGGATGGAGCATCATCAAGATTCACAATTTCTTGGGGCTAGCTGCTTTGCTATTGGCCCTTTGATGATATGGGAATCTTGATGATGCTGCAGCGGCAATAAATGGCTATATATAT

*2G237700_R2*

ACACTCACTCTTCACCCCAATAGCTCATGAATCATGAACATCACACTAAACCATT**CAGGTAAAACATTTAGATACTGGTC**TTTCTTCTTTCTTTTTCTTTTTTGTAATTTTTGAGATGGATGGTGATGGTGAATTTATTAATTATATGAGAAGCACATTGCTGTTTGTTTTGACAATGATGCTTCTTTTCCTATTGGCCTGTTGATTTGTTTTATCTAATGCACCATGTATTTACAATGTTTAATTAAGGATATATATTGGTTTGGGGTTTGTGTTTGTTTGTCTTTGCTTCTTTCTCCCACTTGCACCATTTCGAGTTCTGCAATTGTTCTGTCGTTATCGAGAAAATAAGGGGCGAGTGGGGACTTGGGTTGGGAGGGCAATATCCATTTCATGTGGTAAATACCATTCTGTACTTGTTTAATATATAACTCGAGGTTGTAATTACTTTTAGCTTCGAGCTTTTTCCAGGGAGTGAAGATTTCTCAGAGTTTTGCAGTTCTGGAACTGCAACAGCCCTGATCTCAGGAATGTCTGTACTTTGCTCGAAAATGTTACGCCATATACAAGAAATCATGGGCCTTTTGATTTCGTTTTTTGAAAACTTTCCAAGCACTCtttagatattttctcttttatttatttattattatttggttttttctttTGTTTTTGCTTTTCTAACTCTAGATCCATCACCTTCATGATGGTCTCCATTTTGGAATTACTCTATGCCAGCAGGTTTCAGATTTTGGTGACATGATTTCTTTGGAATATGAGATCAAAGTCTCTCATACAATCATCGCTTGCAGCTGTGTCCAGTTTTGGTTTGTCTGCTTCTCTTTGCTCTCTCTACCACTTTTGTCATGCCGGACAAGACGAGCCCTCATCTACTTATGCCATTCTTTCCCATTTAGAGTTTTTCCAACATTCTTCTTATTTTATCTGTAATCTTTGTGCTCTATCATGTATTAGAAAATATTTTTGATTTCAAGTTTTATTTATCCAACGGGTTGATCTTCGGCGCTGTTGTCATCGCCGTCTAGGGTTTGGAGAAGCCTACTCCATGGGGTAAATGTTATGTGTAATGGCTACTCAAACTCAAATGTATTAGCTCTGTATTTACTCACCTGCTTTGATAAAAACTTTGTAATTGAATCTTTCTTGTTTTGTTCTCTTATTGTTGCGGTCATTTTACTGCTTCTACCACTGCCAGATGTAAAAATTGTGTGAAGTTGTCATTATTTATATCAAAGCTCTATTTTTTGTTGGTCAGTGTGAGCTTCGTCTTATTTGATGTGCTATATAATT

***>pre-miR172d(portion)***

*pre-miR172d_For*

CAAAGACAAAGAGATCCACACAAAAGAAAGACAAAAACCAGTCATTGTTTGCGGATGG**AGCATCATCAAGATTCACAATTTC**TTGGGGCTAGCTGCTTTGCTATTGGCCCTTTGATGATATGGGAA**TCTTGATGATGCTGCAGCGGCA**ATAAATGGCTATATATATACACTCACTCTTCACCCCAATAGCTCATGAATCATGAACATCAC

*pre-miR172d_Rev*
