# Supplementary material for: Less is more: natural variation disrupting a miR172 gene at the di locus underlies the recessive double-flower trait in peach (P. persica L. Batsch)
Source: BMC Plant Biol. 2022 Jul 4;22:318. doi: 10.1186/s12870-022-03691-w (PMC9252053; doi:10.1186/s12870-022-03691-w)
Supplement: Supplementary file 9 — Additional file 9: Supplementary Table S2. Analysis of allelic variant on WGS data retrieved from NCBI SRA database of 41 peach accessions. The presence of di1/di1, di2/di2 and di1/di2 genotypes or of PET alleles in Prupe.6G242400 was assessed. References to phenotype information about double-flower and other ornamental traits are also indicated. Accessions with unclear genotype at either di or Di2 loci were also included. [file 12870_2022_3691_MOESM9_ESM.docx]

| **SRA #** | **Accession** | **Phenotype** | **Genotype** | **Type** | **Origin** | **Note** |
| --- | --- | --- | --- | --- | --- | --- |
| SRR11672390 | Akatsuki | Single | *Di/Di di2/di2* | Improved accession | Japan |  |
| SRR11672326 | Anderson | Single | *Di/Di di2/di2* | Improved accession | Italy |  |
| SRR11672322 | B8-21-20 | Double | *Di/Di di2/di2* | Improved | Henan, PRC | Unclear |
| SRR11672323 | Bai Dan Ban Chui Zhi | Single | *Di/Di di2/di2* | Ornamental | Henan, PRC |  |
| SRR11672387 | Bai Chong Bai Chui Zhi | Double | *Di/Di di2/di2* | Ornamental | Henan, PRC | Unclear |
| SRR11672193 | Bai Hua Shan Bi Tao | Double | *di^1^/di^1^* | Landrace | Beijing, PRC |  |
| SRR14010887 | Bi Tao A | Double | *di^1^/di^1^* | Ornamental |  | peony-form, pink flower |
| SRR14010874 | Bi Tao B | Double | *di^2^/di^2^* | Ornamental |  | peony-form, pink flower |
| SRR11672277 | Bonanza Peach | Single** | *di^1^/di^1^* | Ornamental | USA | **probably double flower |
| SRR11672394 | Changli #1 | Single | *Di/Di di2/di2* | Improved accession | Hebei, PRC |  |
| SRR11672465 | Dan Ban Fen Ju | Single | *Di/Di di2/di2* | Ornamental | Henan, PRC |  |
| SRR11672464 | Dan Ban Hong Ju | Single | *Di/di^2^* | Ornamental | Henan, PRC |  |
| SRR11672406 | Double Delight Nectarine | Single | *Di/di^1^* | Improved accession | USA |  |
| SRR11672205 | Do You | Single | *Di/Di di2/di2* | Improved accession | Japan |  |
| SRR11672130 | Dwarf Norman | Single | *Di/Di di2/di2* | Improved accession | USA |  |
| SRR11672279 | Dwarf Southern Belle Nectarine | Single | *Di/Di di2/di2* | Improved accession | USA |  |
| SRR11672440 | Elegant Lady | Single | *Di/Di di2/di2* | Improved accession | USA |  |
| SRR11672395 | Everts | Single | *Di/Di di2/di2* | Improved accession | South Africa |  |
| SRR11672203 | Favolate #3 | Single | *Di/Di di2/di2* | Improved accession | Italy |  |
| SRR14010876 | Fei Tao | Double | *di^2^/di^2^* | Ornamental | PRC | Upright, red flowers |
| SRR14010897 | Fen Rou Se Bi Tao | Double | *di^2^/di^2^* |  |  |  |
| SRR11672307 | Fen Ling Chong | Double | *PET (Di2/di2)* | Improved | Henan, PRC |  |
| SRR11672386 | Flordacrest | Single | *Di/Di di2/di2* | Improved accession | USA |  |
| SRR11672318 | GF677 | Single | *Di/Di di2/di2* | hybrid | France |  |
| SRR11672159 | Harrow Blood | Single | *Di/Di di2/di2* | Improved accession | Canada |  |
| SRR11672278 | Honey Babe miniature peach | Single | *Di/Di di2/di2* | Improved accession | USA |  |
| SRR14010875 | Hong Bai Chui Zhi | Double | *Di/Di di2/di2* |  |  | Unclear |
| SRR14010764 | Hong Chui Zhi | Double | *PET (Di2/di2)* |  |  |  |
| SRR11672303 | Hong Gen Gan Su Tao | Single | *Di/Di di2/di2* | Wild | Gansu, PRC |  |
| SRR11672392 | Hong Chong Ban | Double | *di^1^/di^1^* | Ornamental | NA |  |
| SRR11672235 | Hong Hua Bi Tao | Double | *di^2^/di^2^* | Landrace | PRC |  |
| SRR11672254 | Hong Hua Chong Ban Chui Zhi 1 | Double | *PET (Di2/di2)* | Ornamental | PRC |  |
| SRR11672306 | Hong Ling Chong | Double | *PET (Di2/di2)* | Improved | Henan, PRC |  |
| SRR11672258 | Hong Shou Xing | Double | *di^1^/di^1^* | Ornamental | Henan, PRC |  |
| SRR11672388 | Hong Ye Chui Zhi | Double | *PET (Di2/di2)* | Ornamental | USA |  |
| SRR11672316 | Hong Ye Tao | Double | *di^1^/di^1^* | Landrace | PRC |  |
| SRR11672161 | Hong Ye Ya Bian | Double | *di^1^/di^1^* | Improved | Henan, PRC | probably from Hong Ye Tao |
| SAMN07215453 | Huang Jin Mei Li | Double | *PET (Di2/di2)* |  |  |  |
| SRR11672305 | Hua Yu Lu | Double | *PET** | Improved | Zhejiang, PRC |  |
| SRR11672363 | Japan #9 | Double | *di^1^/di^1^* | Improved | Japan |  |
| SAMN10584797 | Jiang Tao | Semi-Double | *di^1^/di^1^* | Ornamental | PRC | red flowers (‘Camelliaeflora’) |
| SRR11672336 | Jin Ai Bao Tai | Single | *Di/Di di2/di2* | Improved accession | USA |  |
| SRR11672261 | Ju Hua Tao | Double | *di^1^/di^2^* | Ornamental | Henan, PRC |  |
| SRR11672311 | Li Tao | Double | *di^1^/di^1^* | Ornamental | Japan | Pink Flower |
| SRR11672310 | Li Tao | Double | *di^1^/di^1^* | Ornamental | Japan | Red Flower |
| SAMN07215358 | Man Tian Hong | Double | *di^1^/di^1^* | Ornamental | PRC |  |
| SRR11672244 | Maria Serena | Single | *Di/Di di2/di2* | Improved accession | Italy |  |
| SRR11672315 | NJ271 | Double | *PET (Di2/di2)* | Improved | Henan, PRC |  |
| SRR11672154 | NJC77 | Single | *Di/Di di2/di2* | Improved accession | USA |  |
| SRR11672378 | NJF7 | Single | *Di/Di di2/di2* | Improved accession | USA |  |
| SRR11672131 | NJN76 | Single | *Di/Di di2/di2* | Improved accession | USA |  |
| SRR11672393 | NJ Pillar op R23T62 | Single | *Di/Di di2/di2* | Improved accession | NA |  |
| SRR11672317 | Pegaso | Single | *Di/Di di2/di2* | Improved accession | Italy |  |
| SRR11672241 | Phillips | Single | *Di/Di di2/di2* | Improved accession | USA |  |
| SRR11672242 | Redhaven | Single | *Di/Di di2/di2* | Improved accession | USA |  |
| SRR11672204 | Ri Ben Hong Tian Tao | Single | *Di/Di di2/di2* | Improved accession | Japan |  |
| SRR11672391 | S1 | Double | *di^1^/di^1^* | Ornamental | Beijing, PRC |  |
| SRR11672389 | S2 | Double | *di^1^/di^1^* | Ornamental | Beijing, PRC |  |
| SRR11672259 | Sa Hong Long Zhu Tao | Double | *di^1^/di^1^* | Ornamental | Henan, PRC |  |
| SRR11672260 | Sa Hong Tao | Double | *PET (Di2/di2)?* | Landrace | Henan, PRC |  |
| SRR11672432 | SD-45 | Single | *Di/Di di2/di2* | hybrid | France | *P. davidiana* hybrid |
| SRR11672256 | Shou Bai | Double | *di^1^/di^1^* | Ornamental | PRC |  |
| SRR11672313 | Sunfre | Single | *Di/Di di2/di2* | Improved accession | USA |  |
| SAMN07215375 | Tan Chun | Double | *di^1^/di^1^* |  |  |  |
| SRR11672225 | Tsukuba #86 | Single | *Di/Di di2/di2* | Improved accession | Japan |  |
| SRR11672325 | Venus | Single | *Di/Di di2/di2* | Improved accession | Italy |  |
| SRR11672309 | Wan Chong Fen | Double | *di^2^/di^2^* | Ornamental | Henan, PRC |  |
| SRR11672308 | Wan Chong Hong | Double | *di^2^/di^2^* | Ornamental | Henan, PRC |  |
| SAMN07215616 | Wu Bao Tao | Double | *di^2^/di^2^* | Ornamental | PRC | Upright |
| SAMN10584785 | Ying Chun | Double | *di^1^/di^1^* | Ornamental | PRC |  |
| SAMN10584831 | Yuan Chun | Double | *di^1^/di^1^* | Ornamental | PRC |  |
| SRR11672252 | Yuan Yang Chui Zhi | Double | *di^1^/di^1^* | Ornamental | PRC |  |
| SRR11672240 | Zhong You Pan #2 | Single | *Di/Di di2/di2* | Improved accession | Henan, PRC |  |
| SRR11672253 | Zhu Fen Chui Zhi | Double | *di^1^/di^1^* | Ornamental | PRC |  |
